# Tethered optoacoustic and optical coherence tomography capsule endoscopy for label-free assessment of Barrett’s oesophageal neoplasia

**DOI:** 10.1038/s41551-025-01462-0

**Published:** 2025-08-06

**Authors:** Qian Li, Zakiullah Ali, Christian Zakian, Massimiliano di Pietro, Judith Honing, Maria O’Donovan, Krzysztof Flisikowski, Vassilis Sarantos, Guillaume Pierre, Jerome Gloriod, Wolfgang Drexler, Vasilis Ntziachristos

**Affiliations:** 1 Center for Medical Physics and Biomedical Engineering, Medical University Vienna, Vienna, Austria; 2 Chair of Biological Imaging, Central Institute for Translational Cancer Research (TranslaTUM), School of Medicine and Health & School of Computation, Information and Technology, Technical University of Munich, Munich, Germany; 3 Institute of Biological and Medical Imaging, Bioengineering Center, Helmholtz Zentrum München, Neuherberg, Germany; 4 Early Cancer Institute, University of Cambridge, Cambridge, UK; 5 Department of Histopathology, Cambridge University Hospital, Cambridge, UK; 6 Chair of Livestock Biotechnology, School of Life Sciences, Technical University of Munich, Munich, Germany; 7 RayFos: Scientific Software, Basingstoke, UK; 8 SONAXIS S.A., Besançon, France; 9 Statice, Besançon, France; 10 Munich Institute of Robotics and Machine Intelligence (MIRMI), Technical University of Munich, Munich, Germany; 11 Munich Institute of Biomedical Engineering (MIBE), Technical University of Munich, Garching b. München, Germany

**Keywords:** Gastrointestinal cancer, Cancer imaging, Oesophageal diseases

## Abstract

Endoscopic detection of oesophageal cancer (EC) often occurs late in disease development, leading to high mortality rates. Improved technologies are urgently needed for earlier EC detection. Here we research an endoscopic ultra-broadband acoustic detection scheme and introduce a 360-degree hybrid optoacoustic and optical coherence endoscopy to enable interrogation of surface and subsurface precancerous and cancerous features at a three-dimensional micrometre scale. In the following pilot tissue investigation, the dual-modal imaging features are assessed for classifying different mucosal types in Barrett’s oesophagus (BE)—a precursor of EC. We find that human lesions of different grades, such as metaplastic, dysplastic and cancerous mucosa, exhibit distinctly different imaging features that are unique to the hybrid modality. Based on these features, a classification system is developed and evaluated for identifying BE neoplasia. The results show accurate BE neoplasia detection due to the complementarity of the two imaging modalities. Therefore, this study highlights the ability of the new dual-modality feature set to improve the detection performance of any of the two modalities operating in stand-alone mode and enhance diagnostic accuracy.

## Abstract (web summary)

A high-resolution three-dimensional endoscopic imaging technique resolves unique features in Barrett’s oesophageal neoplasia.

## Main

Oesophageal cancer (EC) affects more than 600,000 people annually worldwide^1^. Oesophageal adenocarcinoma (EAC) is the predominant EC type in western countries^2^ and its incidence has dramatically increased in the past three to four decades^3^. Risk factors for EAC, including gastro-oesophageal reflux disease, obesity and Barrett’s oesophagus (BE), are estimated to implicate over 300 million people in the world^4,5^. Despite advances in its treatment, EC is a deadly cancer with the second lowest survival rate of all cancers^6^. A driving reason for the high mortality relates to its late detection. EAC is detected today based on white light endoscopy (WLE) and analysis of biopsies taken from the oesophageal lumen^7,8^. However, early neoplasia, including dysplasia (a precancerous lesion) and early-stage cancer, is often difficult to distinguish under WLE, even with the presence of BE^9^—the only known precursor of EAC^10^. As a result, guidelines advise four-quadrant random biopsies within BE mucosa to find focal neoplasia^7,11^. Despite the considerable time and expense involved^11^, this systematic biopsy protocol samples only a small subset of BE^12,13^ and misses 30% (refs. ^14,15^) to 50% (refs. ^16,17^) of early neoplastic lesions. Conversely, when detected at the early stage, EAC can be effectively treated before progressing to invasive cancers^18^.

Many imaging techniques have been considered for improving endoscopic performance. Chromoendoscopy uses a variety of dyes such as methylene blue or acetic acid to improve visualization of architectural details of mucosa and highlight abnormal areas. Narrow band imaging illuminates the tissue surface with blue and green light to enhance microvasculatures and mucosal contrast. Confocal laser endomicroscopy visualizes small fields of view of mucosa with high resolutions, allowing assessment of cellular and subcellular structures in vivo. Several studies have demonstrated the clinical potential for these methods^9,19–21^. However, the use of confocal laser endomicroscopy is limited by its small field of view^22^. Chromoendoscopy is not routinely employed either, as it prolongs examination time and its detection benefit is debated^23,24^. Narrow band imaging has become part of the examination process in many endoscopy suites but it has not demonstrated substantial advantages in neoplasia detection^15,25,26^. Furthermore, a critical limitation of these approaches is that they only capture superficial contrast with depths that generally do not exceed 100 µm.

Optical coherence tomography (OCT) and optoacoustic imaging (OAI) are two naturally complementary optical imaging technologies in terms of contrast mechanism, penetration depth and spatial resolution. OCT primarily visualizes morphological features that relate to differences in refractive index generated by tissue microlesion interfaces. The method has shown detailed imaging of oesophageal wall layers up to a depth of ~2.5 mm (refs. ^27,28^). Various endoscopic OCT devices, such as line-scanning catheter^27–29^, balloon catheter^30,31^, micromotor catheter^32^ and tethered capsules^33–36^, have been developed and evaluated in surveillance of patients with BE. By means of sensing blood flow, endoscopic OCT angiography (OCTA) may further reveal vascular features within the oesophageal mucosa^37,38^. However, OCT has not yet met the diagnostic thresholds for replacing the current standard^39^, especially since detection of early EAC still remains challenging^40^. In contrast to OCT, OAI is capable of non-invasive visualization of optical absorbers within tissues, offering a fundamentally different contrast mechanism^41,42^. For gastrointestinal tract applications, visualization of intricate mucosal vascular morphology has been demonstrated using different optoacoustic endoscopy systems^43–50^, but mainly in healthy animal models and with bandwidths at the few tens of megahertz, leading to resolutions of several tens of micrometres. Conversely, higher bandwidth (~100 MHz) ultrasound detectors improve the OAI resolution to a few tens of micrometres or better, especially at superficial depths, matching the oesophageal-wall depth dimension. The resultant optoacoustic mesoscopy^51^ (OPAM), based on such broadband detectors, offers a resolution-to-depth ratio beyond other optical modalities. Within the depth regime of a few millimetres necessary for imaging of oesophageal neoplasia, the resolutions of OPAM are comparable to OCT, making OPAM ideal for delivering a different source of contrast to complement the OCT contrast. The clinical use of OPAM has been demonstrated in psoriasis^52^ and melanoma^53^ but its performance in imaging oesophageal lumens is not yet known.

Here we explore the contrast that can be achieved by OPAM in mucosal imaging and investigate the merits of hybrid OCT and OPAM endoscopy (O2E) in resolving neoplastic features in BE. Through a 5-year development programme, spearheaded by the European Union grant ESOTRAC, we developed a unique 12.5-mm-diameter tethered capsule that encloses both OCT and OPAM modalities, enabling label-free, depth-resolved cross-sectional and en face oesophageal imaging. To enable O2E, we researched an ultrasound detection concept that allows miniaturized, ultra-broadband (100 MHz) endoscopic OPAM detecting at 90° angle, in relation to the endoscope axis, with illumination through the detector for OCT and optoacoustic signal excitation. This development was followed by pilot tissue investigations aiming to assess O2E imaging features for classifying metaplastic, dysplastic and cancerous mucosae within BE. We identified unique O2E imaging features interpretable by clinical and non-clinical users and used them to develop and validate a user-friendly classification system to identify BE neoplasia. The results show first, that OPAM provides key features that are representative of early cancer and not available to OCT. Second, the O2E modality offers unprecedented complementary features compared to stand-alone OCT or OPAM. Thus, the combination of OCT and OPAM is essential for distinguishing neoplasia in BE. We discuss that O2E holds translational potential to become an endoscopic approach with superior three-dimensional (3D) detection ability of neoplasia in BE mucosa.

## Results

### Development of O2E capsule with ultra-broadband transducer

**Fig. 1:**
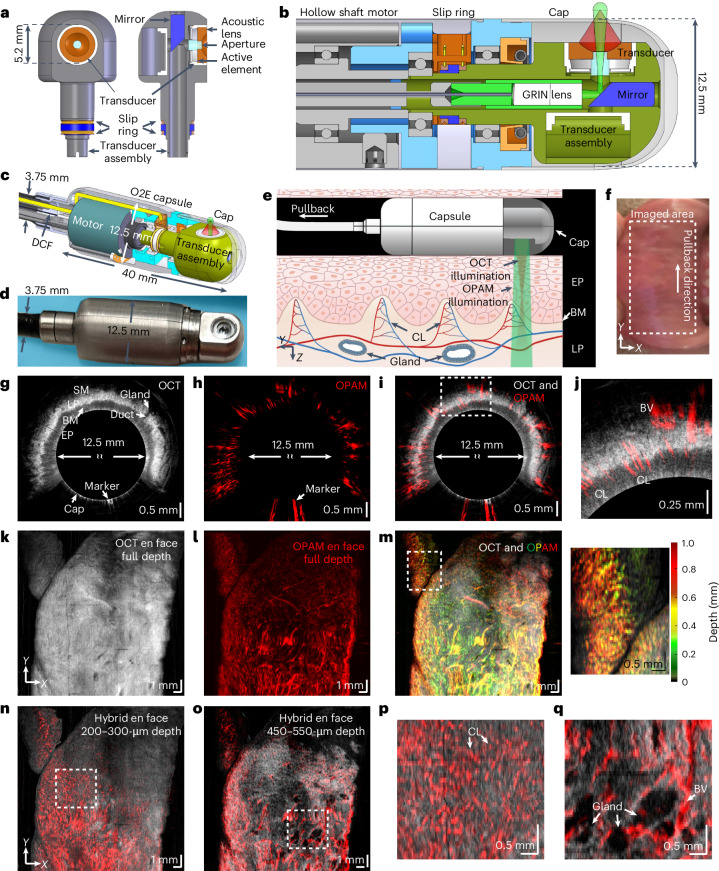
Tethered O2E capsule and its imaging of human oral mucosa. **a**, En face and cross-sectional views of the developed ultra-broadband hollow transducer with a central aperture of 1.2 mm for OPAM/OCT light to pass through. **b**, Cross-sectional view of the O2E capsule. The motor rotates the transducer assembly to generate circumferential imaging. The GRIN lens is kept static inside the hollow shaft of the transducer assembly. **c**, Cross-sectional view of a 3D model of the O2E capsule. **d**, Photograph of the O2E capsule without the cap. **e**, Schematic diagram showing the principle of pulling back the O2E capsule to scan a segment of squamous mucosa. The capsule needs to be in contact with the mucosa for imaging. **f**, Photograph of the oral mucosa imaged by O2E. **g–i**, OCT (**g**), OPAM (**h**) and hybrid of OCT and OPAM (**i**) cross-sectional images of the oral mucosa. A marker attached at the cap surface to align consecutive cross-sectional images can be seen. **j**, Magnified view of the dashed box in **i** shows small superficial capillary loops (CL) perpendicular to the mucosal surface and large blood vessels (BV) inside lamina proprial/submucosa (LP/SM) layers. **k–m**, Full-depth projection OCT (**k**), OPAM (**l**) and hybrid (**m**) en face images of the oral mucosa in Cartesian coordinates. OPAM image is depth-coded in **m** to show vascular morphology and vascular depth distributions simultaneously. **n**,**o**, Hybrid subsurface en face images of the oral mucosa at 200–300 µm (**n**) and 450–550 µm (**o**) depths. Visualization at these depths reveals marked differences in mucosal and vascular architectures between EP and LP/SM layers. **p**,**q**, Magnified views of the dashed boxes in **n** (**p**) and **o** (**q**). BM, basement membrane.

A key element for O2E is a hollow, spherically focused, ultra-broadband lithium niobate ultrasound transducer (Fig. 1a) researched in this study. The transducer sits inside a transducer assembly (Fig. 1a) with customized gold slip rings to transmit OPAM signals. A 45° mirror inside the transducer directs OCT/OPAM light through a 1.2-mm-diameter aperture for side illuminations.

The miniaturized transducer enabled the development of an O2E capsule (Fig. 1b–d) delivering 30-Hz framerate, high-resolution endoscopic imaging. The 40-mm-long, 12.5-mm-diameter capsule was encapsulated with a transparent cap. A motor inside the capsule rotates the transducer assembly for 360° circumferential imaging. Pulling back the capsule from lumen organs, while the motor rotates, generates a helical scan of the tissue investigated. A double-clad fibre (DCF) propagates the 532-nm OPAM photon pulses and 1,060-nm broadband OCT light through the hollow shaft of the motor to the capsule. A gradient-index (GRIN) lens co-axially focuses the OCT light at 200 µm inside the tissue and irradiates the tissue surface divergently with a 3.9° half angle for OPAM (Fig. 1e). The 532-nm wavelength of OPAM was selected due to its high absorption by haemoglobin, leading to strong contrast in visualizing microvascular morphology. The OCT wavelength was selected to operate at the low absorption window by the OPAM coupling medium (deuterium oxide) inside the capsule, while yielding strong OCT contrast^36^.

The transducer developed for OPAM is based on a previously characterized technology, featuring a lithium niobate-based ultrasound transducer exhibiting 100-MHz bandwidth at −6 dB cut-off frequency, allowing 30-µm lateral and 17-µm axial resolution, comparable to the 10-µm lateral and 7-µm axial resolution of the OCT. Selecting the high-frequency components in optoacoustic signals for OPAM reconstruction can report higher resolution, that is the OPAM images reconstructed at a selected high-frequency band (that is 40–110 MHz) yields resolutions close to those of OCT. A detailed description of the O2E capsule, the system setup, characterization and OPAM reconstruction can be found in Methods.

### In vivo and ex vivo O2E imaging of healthy mucosa

The basic imaging feasibility of the O2E system was demonstrated by scanning a healthy human oral cavity and a swine oesophagus. For human imaging, a volunteer wrapped his labial mucosa (Fig. 1f) around the capsule and performed a motorized helical scan (20-mm longitudinal pullback) (see Methods for ethical approval and workflow). The OCT image obtained (Fig. 1g) showcases a typical stratified architecture of the labial mucosa, whereby the squamous epithelium, lamina propria and the basement membrane separating these two layers can be distinguished due to the relative differences in the intensity of their back reflections. Microstructures in the submucosa, including salivary glands and their ducts, are clearly seen as poorly scattering voids and tubes, respectively. Consistent with previous studies^54,55^, the epithelium is hypo-reflective in OCT, while the lamina propria and submucosa are hyper-reflective.

Cross-sectional OPAM images (Fig. 1h) resolved mucosal vasculatures not visible in OCT structural imaging and allowed observation of depth-dependent vascular morphology revealing superficial capillary loops (Fig. 1j) perpendicular to the surface and larger blood vessels (Fig. 1j) lying within the lamina propria and submucosa. Different OPAM images can be reconstructed at separate frequency bands to visualize distinct features^52^. Supplementary Note 3 shows that images at the low-frequency band (3–40 MHz) selectively visualize large vessels as they generate lower ultrasound frequencies, whereby images at the high-frequency band (40–110 MHz) highlight capillary loops and small vessels. Frequency-band separation allows better visualization of the finer features by eliminating their possible masking by the much higher-intensity low-frequency components^52^ and results in a better representation of both larger and smaller features.

Macroscopic mucosal and vascular architectures can also be seen on en face images (Fig. 1k–q). OCT full-depth projection en face image (Fig. 1k) primarily displays a smooth mucosa. Conversely, subsurface en face images unfold a strong contrast between the uniform epithelial layer (Fig. 1n) and the glandular deeper layers (Fig. 1o). A depth-resolved OPAM en face image (Fig. 1m) uncovers a depth-dependent vascular pattern with dotted superficial capillary loops (Fig. 1n,p) residing in the papillae and mesh-like deeper vascular networks (Fig. 1o). Observation of hybrid images, combining OCT and OPAM (Fig. 1q), reveals that the mesh-like vascular pattern seen on the OPAM images is due to microvasculature that encircles glands (Fig. 1q). Details regarding the generation of OCT and OPAM en face images can be found in Methods.

The in vivo O2E imaging performance demonstrated on healthy human mucosa was corroborated by ex vivo and in vivo imaging of a swine oesophagus (see Methods for ethical approval and workflow). The study confirmed that OCT resolved the subsurface oesophageal layers (Supplementary Notes 4 and 5) and that OPAM provided enhanced microvascular visualizations (Supplementary Note 5). Ex vivo imaging of the swine oesophagus (Supplementary Note 4) served as a negative control for OPAM, due to the decrease in haemoglobin content in the blood vessels of excised tissue. The ex vivo OPAM images (Supplementary Fig. 4) showcase reduced contrast, confirming that the mucosal contrast visualized on the in vivo images is primarily due to haemoglobin and microvasculatures.

### Ex vivo O2E imaging of human BE specimens and validation

**Fig. 2:**
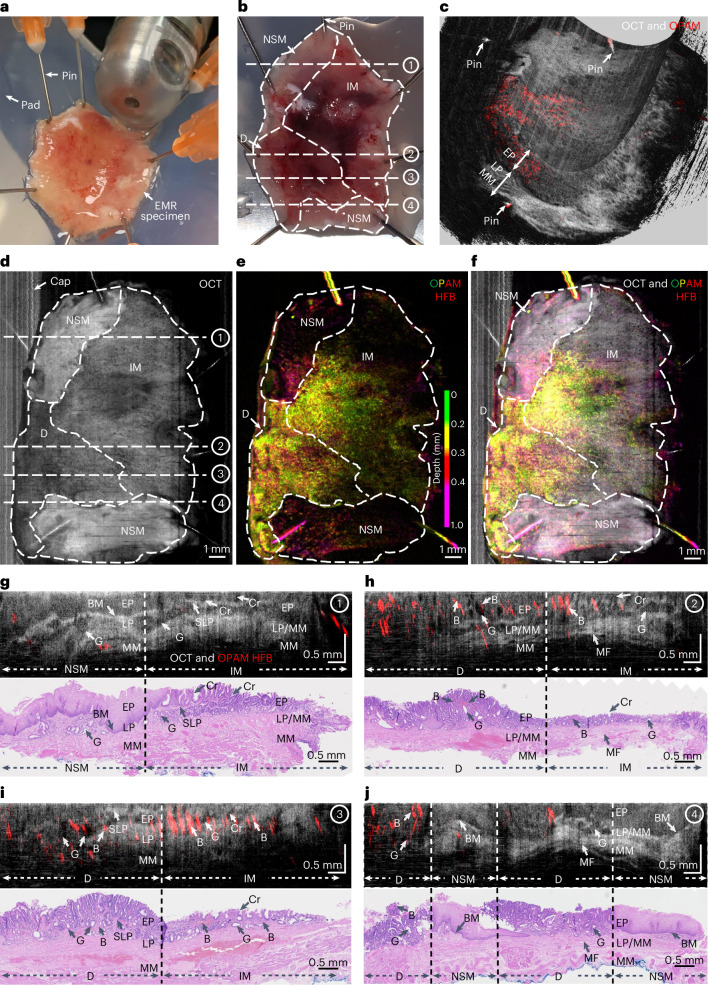
O2E imaging of EMR specimens. **a**, Photograph showing an EMR specimen fixated on a flexible pad with O2E capsule ready to scan the specimen. **b**, Histopathological mapping imposed over a photograph of a specimen. Each specimen was divided into separate regions based on the respective histopathological types. These regions serve as ROIs for analysis in this study. NSM, normal squamous mucosa; IM, intestinal metaplasia; D, dysplasia. **c**, Cropped 3D-rendered O2E visualization of the specimen in cylindrical coordinates. **d**, OCT full-depth projection en face image of the specimen in **b**. In areas without contact to the specimen, strong optical reflection from the cap can be seen. **e**, Depth-resolved OPAM en face image of the specimen reconstructed with high-frequency band (40–110 MHz) components. **f**, Hybrid en face image merging OCT and OPAM HFB. **g–j**, Representative O2E cross-sectional images with correlated H&E stained histopathology slides showing clear contrasts between NSM and IM in **g**, clear divisions between IM and D in **h** and **i**, and distinct differences among local regions of NSM, IM and D in **j**. Image locations are illustrated as dashed lines in **b** and **d**. B, blood; Cr, crypt; G, gland; HFB, high-frequency band; LP/MM, mixture of lamina propria and muscularis mucosae; MF, muscle fibre; MM, muscularis mucosae; SLP, superficial lamina propria.

Following the imaging validation studies, we aimed to investigate the BE features resolved by O2E. For this purpose, a pilot clinical study (see Methods for ethical approvals and workflow) was conducted to image endoscopic mucosal resection (EMR) specimens from patients with BE. A total of 14 specimens (average size: 2 cm^2^) suspected of BE neoplasia were resected from 10 patients and included in this study. The specimens were pinned on flexible pads to mimic oesophageal lumen for imaging (Fig. 2a). After imaging, the specimens were sectioned and stained (Methods) for a thorough histopathological analysis. An expert gastrointestinal pathologist assessed the sectioned slides on a millimetre-per-millimetre basis and classified each millimetre-cell of mucosa into five categories: normal squamous mucosa, gastric metaplasia, intestinal metaplasia, dysplasia and cancer. The specimens were then partitioned into regions corresponding to the mucosal types. Figure 2b showcases the histopathological segmentation of a specimen into two regions of normal mucosa, one region of intestinal metaplasia and one region of dysplasia. Histopathological segmentation of all specimens resulted in a total of 61 regions of interest (ROIs) comprising 9 normal ROIs, 13 gastric metaplastic ROIs, 12 intestinal metaplastic ROIs, 19 dysplastic ROIs and 8 ROIs of intramucosal cancer (stage T1a). As growing evidence^56–59^ suggests gastric metaplasia as the likely precursor of intestinal metaplasia, these ROIs cover all stages in the progression from metaplasia to dysplasia, and then to early cancer.

To establish a one-to-one correlation between O2E imaging and histopathology, the fixation pins employed for O2E imaging (Fig. 2b,c) were used as markers due to their visibility in both modalities. The pathologist took note of relative distances of each section to these pins. For the correlation, O2E images were converted from cylindrical (Fig. 2c) to Cartesian coordinates (Fig. 2d–f). The findings demonstrated high-quality correlation between histopathology and O2E (Fig. 2g–j). In regions of normal squamous mucosa, mucosal layers including squamous epithelium, lamina propria and muscularis mucosae can be clearly distinguished in OCT (Fig. 2g,j). Similar to labial mucosa, an intact basement membrane can be seen in normal oesophageal mucosa to separate the epithelium and lamina propria. OCT resolves a typical crypt architecture in intestinal metaplastic mucosa with crypts shown as low-scattering superficial tubular structures (Fig. 2g,h). Dysplasia presents a more disorganized crypt architecture with crowded superficial glands in histology. In OCT, dysplastic glands are shown as low-scattering voids, and they correspond well to histology (Fig. 2h–j). Layers of epithelium, lamina propria and muscularis mucosae can be observed in some intestinal metaplastic and dysplastic areas (Fig. 2h,j). Superficial lamina propria—a feature of BE mucosal remodelling^60^—can be visualized in OCT images of intestinal metaplasia and dysplasia (Fig. 2g,i). Likewise, OPAM features correspond well to vasculatures, based on contrast from blood that remained in the microvasculatures of the histopathological slides (Fig. 2h–j). An analysis of OPAM features and corresponding features from CD31 immunostained sections (Methods) demonstrated a good correlation between the two modalities (Supplementary Note 8).

### Analysis of cross-sectional O2E image features of BE

**Fig. 3:**
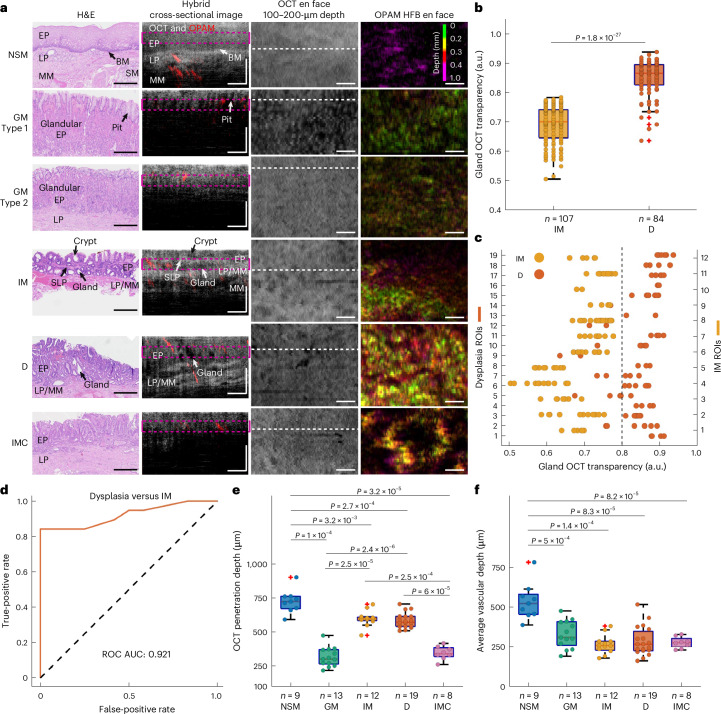
O2E features observed in cross-sectional images of different mucosal types. **a**, Representative H&E, hybrid cross-sectional, OCT 100–200-µm depth projection and OPAM HFB en face images of NSM, gastric metaplasia (GM) type 1, GM type 2, IM, D and intramucosal cancer (IMC). White dashed lines correspond to the locations of cross-sectional images in en face images. Pink dashed lines in hybrid cross-sectional images indicate the 100–200-µm depth range for visualizing the epithelial patterns in en face images. Scale bars, 0.5 mm. **b**, Box and whisker plots showing the OCT transparency of intestinal metaplastic and dysplastic glands retrieved from IM and dysplastic ROIs. **c**, Scatter plot showing the OCT transparency of glands extracted from IM and dysplastic ROIs. The dashed line corresponds to the OCT transparency index value of 80%. **d**, ROC curve generated using the average gland OCT transparency of an ROI as the marker to distinguish dysplastic ROIs (*n* = 19) and IM ROIs (*n* = 12). **e**, Box and whisker plots of quantified OCT penetration depth in NSM, GM, IM, D and IMC ROIs. **f**, Box and whisker plot showing the calculated average depth of imaged vasculatures in NSM, GM, IM, D and IMC ROIs. All quantities with significant statistical differences (5% significance value) have been indicated in this figure. The differences between samples were estimated using two-sided Wilcoxon rank sum tests. On each box in the box and whisker plots, the central mark indicates the median, and the bottom and top edges of the box indicate the 25th and 75th percentiles, respectively. The whiskers extend to the most extreme data points not considered outliers, and the outliers are represented by ʽ+ʼ. Source data

The detailed histopathological validation, described in the previous section, allows us to summarize key O2E imaging features associated with the BE mucosa. Focusing first on features on cross-sectional images (Fig. 3a), we observed a clear mucosal stratification with a uniform avascular epithelial layer, a high-scattering lamina propria layer and an apparent basement membrane separating the two layers (Fig. 3a) in normal squamous mucosa. Conversely, intestinal metaplasia and dysplasia were characterized by surface crypts and underlying glands in their epithelial compartments^61^. Crypts and glands are recognized as low reflectance tubular structures and voids in OCT, respectively. In intestinal metaplasia, the epithelium is interlaced with high-scattering connective tissues and low-scattering crypts or glands (Fig. 3a). This pattern is not common in dysplasia due to reduced intervening lamina propria^61^ in its epithelium. A prominent feature of dysplasia is the crowded epithelial glands with much lower OCT signals in the gland lumen than intestinal metaplasia (Fig. 3a). A quantitative comparison of OCT intensities in dysplastic glands and intestinal metaplastic glands was performed by extracting OCT signals within these gland lumens and normalizing the signals using a custom OCT transparency index (Methods). The derived index reflects the degree of transparency of the gland lumen to the OCT light. Results show significantly higher OCT transparency (*P* < 0.0001) of dysplastic glands than that of intestinal metaplastic glands (Fig. 3b). Furthermore, the existence of transparent glands in OCT (≥80% transparency) is a hallmark of dysplasia as 84% of dysplastic ROIs contain at least one of such glands versus 0% of intestinal metaplasia (Fig. 3c). Using the averaged gland transparency within an ROI (Methods) as the marker to differentiate intestinal metaplastic and dysplastic ROIs demonstrates an outstanding discriminatory power with an area under the receiver operating characteristic curve (AUROC) of 0.92 (Fig. 3d). Dysplasia may also showcase a loss of a clear mucosal stratification in OCT. We observed a clear stratification in 26% cross-sectional images of dysplasia compared to 75% of intestinal metaplasia.

Distinctive from other types, gastric metaplasia and intramucosal cancer both exhibit a markedly reduced penetration and a complete effacement of mucosal stratification in OCT (Fig. 3a). Quantification of the depth reached by OCT (Methods) in all types of ROIs shows significantly lower penetration in gastric metaplasia and intramucosal cancer than the other types (Fig. 3e), but without a significant statistical difference (*P* = 0.26) between gastric metaplasia and intramucosal cancer. For gastric metaplasia with prominent pits, OCT clearly visualizes the vertical pit architecture (Fig. 3a). However, in gastric metaplasia with short pit compartments, OCT image shows a homogenous subsurface architecture similar to intramucosal cancer (Fig. 3a). We annotate gastric metaplasia with prominent superficial pits as gastric metaplasia type 1 and the other as type 2 in this study. Except for the distinct configurations of their pit compartments, gastric metaplasia type 1 and 2 display similar histological features that resemble normal gastric mucosa.

OPAM corroborates these findings by resolving vasculatures within BE mucosa. Cross-sectional images of different mucosal types in BE (Fig. 3a) reveal strikingly different vascular distribution patterns between normal and abnormal mucosae, the latter including metaplasia, dysplasia and intramucosal cancer. The normal mucosa only contains deep vasculatures beyond the epithelium, while the columnarized abnormal mucosae are densely vascularized in their epitheliums (Fig. 2g–j and Fig. 3a). Quantifications of the depths of imaged blood vessels in all ROIs (Methods) demonstrate significantly (*P* < 0.001) shallower vascular depth (Fig. 3f) in abnormal ROIs than normal ones. Although dense epithelial vasculatures often block OPAM illumination from reaching deeper tissue, correlating OPAM with CD31 immunostained images in Supplementary Note 8 shows imaging of deep vasculatures at a depth of ~1.5 mm in the mucosa. Based on the good correlation between OPAM and CD31, we showcase in Supplementary Note 9 that OPAM accurately reconstructs blood vessel cross sections. For vessels oriented perpendicularly to the cross-sectional imaging plane, the cross-sectional images show the transverse sections of vessels as round dots. Otherwise, the oblique sections of vessels are displayed in the form of ellipses. Vessel diameters determined in OPAM were found to match the histologically determined values (Supplementary Note 9). Measurement of major and minor axis length of the elliptic oblique vessel section in OPAM allows estimation of vessel orientation (Supplementary Note 9).

### Analysis of en face O2E image features of BE

**Fig. 4:**
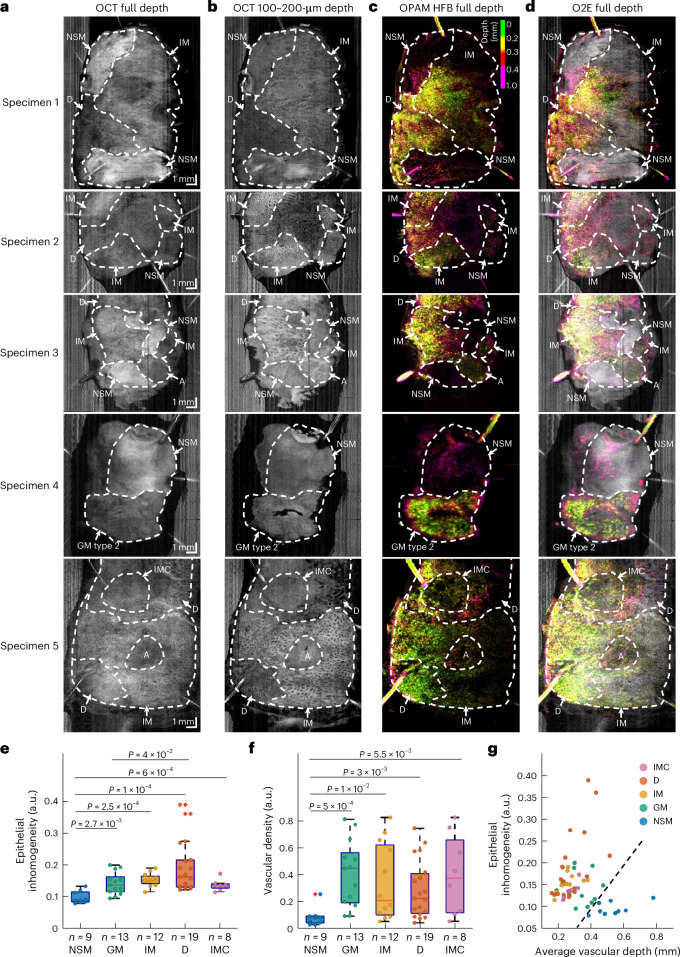
O2E en face images reveal distinct epithelial and vascular patterns in abnormal regions. **a–d**, Representative OCT full-depth projection (**a**), OCT 100–200-µm depth projection (**b**), OPAM HFB (**c**) and O2E full-depth projection (**d**) en face images of five EMR specimens. Area A shows regions affected by artefacts. In these regions the epitheliums have been damaged so that the exact histopathological mucosal type cannot be determined. **e**, Box and whisker plot showing the calculated epithelial inhomogeneity in NSM, GM, IM, D and IMC ROIs. **f**, Box and whisker plots showing the quantified vascular density in NSM, GM, IM, D and IMC ROIs. **g**, Illustrative scatter plot of the calculated epithelial inhomogeneity versus average vascular depth for all 61 ROIs. These two features (epithelial inhomogeneity and average vascular depth) cluster normal and abnormal (GM, IM, D and IMC) ROIs. The black dashed line indicates that a perfect separation of abnormal ROIs from normal ones can be achieved based on these features. All quantities with significant statistical differences (5% significance value) have been indicated in this figure. The differences between samples were estimated using two-sided Wilcoxon rank sum tests. On each box in the box and whisker plots, the central mark indicates the median, and the bottom and top edges of the box indicate the 25th and 75th percentiles, respectively. The whiskers extend to the most extreme data points not considered outliers, and the outliers are represented by ʽ+ʼ. Source data

Observation of full-depth projection en face OCT images (Fig. 4a), generated as average intensity projections along the depth axis for the entire 3D data volume, was found to average different types of mucosae, challenging the identification of individual features. However, en face OCT images generated at 100–200-µm depths (Fig. 4b) reveal a stark contrast in epithelial patterns between normal and abnormal (metaplastic, dysplastic and cancerous) mucosae. While normal oesophageal mucosa displays a uniform epithelium, abnormal mucosae show a markedly different, inhomogeneous epithelium in OCT. Quantification of the observed epithelial inhomogeneity in OCT (Methods) demonstrates significant differences between normal and abnormal ROIs (Fig. 4e). An increasing in grades from normal to metaplasia and then dysplasia is associated with a rising epithelial inhomogeneity (Fig. 4e). However, gastric metaplasia and intramucosal cancer are not statistically different (*P* = 0.86) in their epithelial inhomogeneity (Fig. 4e).

High-frequency band OPAM en face images (Fig. 4c,d) are shown with a depth-coding scheme, which renders epithelial vessels found at depths of 0–0.3 mm in green and yellow and vessels deeper in tissue in red and magenta. The coding scheme remarkably highlights the abnormal mucosae due to the densely vascularized columnar epithelium observed in cross-sectional images. Quantification of the vascular density according to OPAM (Methods) demonstrates significantly (*P* < 0.001) denser vasculatures in abnormal mucosae than normal ones (Fig. 4f). Therefore, en face OCT images at 100–200-µm depths and depth-coded en face OPAM images enable direct visual detection of abnormal areas within BE. Furthermore, the distribution of calculated average depth of blood vessels and epithelial inhomogeneity in all 61 ROIs (Fig. 4g) illustrates that this feature set clusters the normal and abnormal ROIs separately, suggesting that normal and abnormal mucosae can be objectively distinguished based on these two features. Supplementary Note 10 showcases that as the amplitude of low-frequency components in OPAM signals is significantly higher than that of high-frequency components, it is essential to use only the high-frequency band for image reconstruction to resolve fine vascular features in BE.

**Fig. 5:**
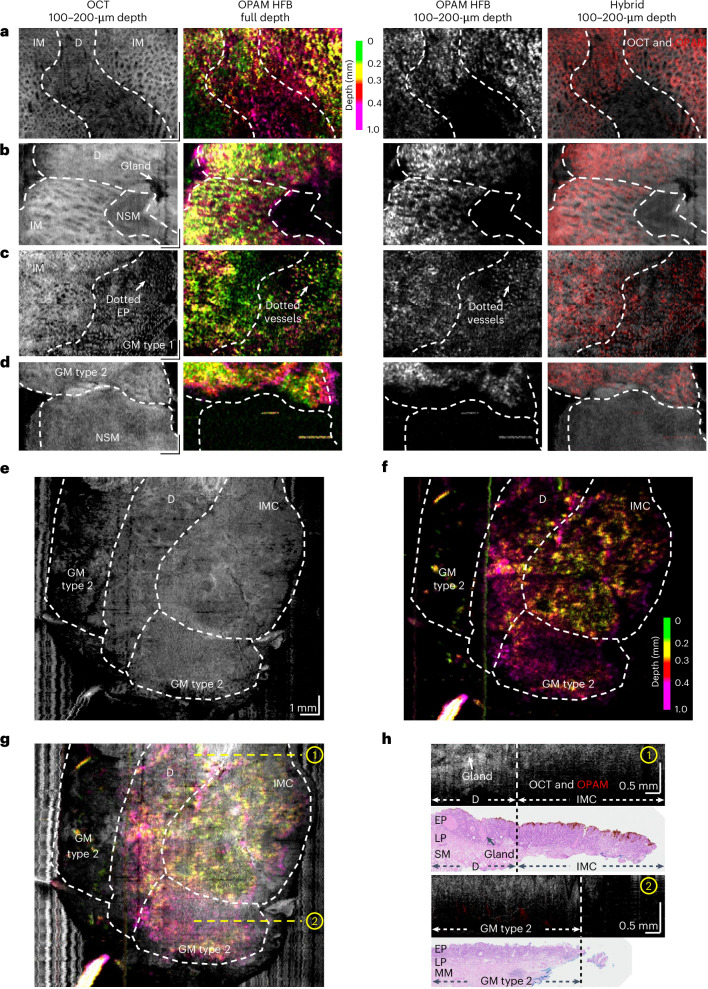
En face O2E features of different mucosal types. **a–d**, representative OCT 100–200-µm en face images (**a**), OPAM HFB full-depth en face images (**b**), OPAM HFB 100–200-µm en face images (**c**) and hybrid 100–200-µm en face images (**d**) of NSM, GM, IM and D show distinct en face features in different types of mucosae. Scale bars, 1 mm. **e**, OCT 100–200-µm en face image of an EMR specimen containing a region of IMC. **f**, OPAM full-frequency band (FFB) full-depth en face image of the cancerous specimen shows a typical tumour vascular pattern with dilated superficial vessels forming meandering loops. **g**, Hybrid OCT and OPAM FFB full-depth en face image. **h**, Cross-sectional hybrid and H&E images of the cancerous EMR specimen at locations indicated by dashed yellow lines in **g**. The dysplastic region shows an larger OCT penetration depth than IMC with the presence of dysplastic glands.

En face images also uncover specific morphological features for different mucosal types. OCT 100–200 µm and OPAM high-frequency band en face images unveil honeycomb vascular and epithelial pattern in intestinal metaplasia (Fig. 5a–c), as well as irregular epithelial morphology with atypical glands and irregular vascular patterns in dysplasia (Fig. 5a,b). Gastric metaplasia type 1 shows a stippled epithelial pattern in OCT and dotted epithelial vasculatures in OPAM due to its prominent pit architectures (Fig. 5c). As a comparison, the epithelium of gastric metaplasia type 2 displays in OCT a relatively uniform pattern without well-characterized structures (Fig. 5d,e). We demonstrate that OPAM is essential for detecting intramucosal cancer. With OCT alone, distinguishing intramucosal cancer and gastric metaplasia type 2 is challenging as they both present a reduced penetration and a structureless homogeneous mucosa in either en face images (Figs. 4b and 5e) or cross-sectional images (Fig. 5h). Relying on OPAM, typical tumour superficial vascular patterns^62,63^ with dilated vessels forming meandering loops around avascular areas are revealed to provide the vital information for identifying either a small (~3 × 3 mm^2^, Fig. 4c) or a larger (~7 × 7 mm^2^, Fig. 5g) region of intramucosal cancer. Figure 5h again shows clear differences between intramucosal cancer and dysplasia with irregular glands in dysplasia versus a significantly reduced OCT penetration in the adjacent cancerous mucosa.

### Development and validation of O2E classification system

**Fig. 6:**
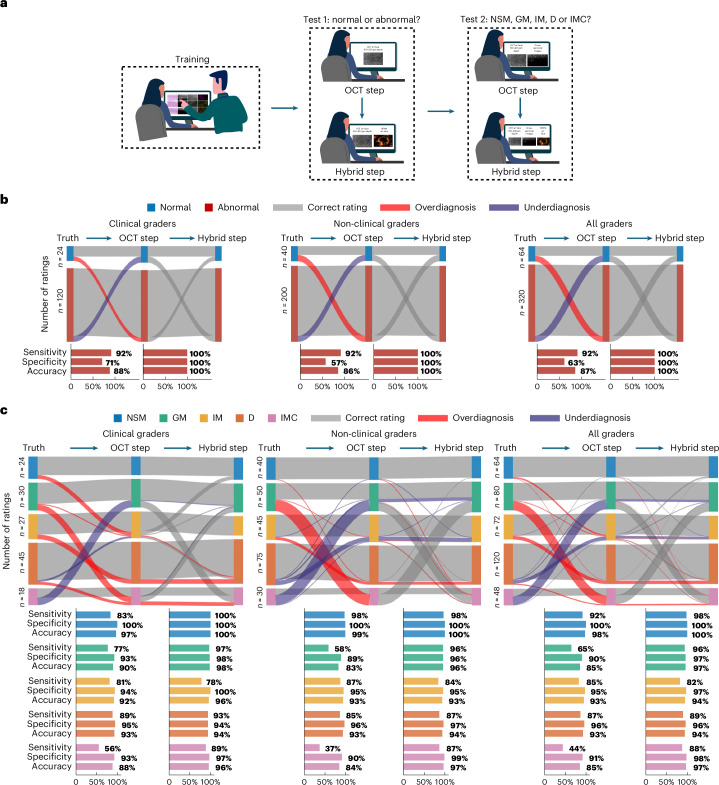
Development and validation of O2E classification system. **a**, Schematic overview of the procedures of classification tests. Classification tests were conducted to validate the effectiveness of O2E features for identifying different types of mucosae in BE. Eight graders were first trained in O2E features and then enrolled to two classification tests. In Test 1, graders individually classified 48 ROIs as either normal or abnormal mucosa. Abnormal mucosa refers to GM, IM, D or IMC. In Test 2, graders classified 48 ROIs as NSM, GM, IM, D or IMC. Each test contains two steps: OCT step and subsequent Hybrid step. Ratings made in OCT step were based on OCT images. In Hybrid stage, ratings were made based on OCT and OPAM images side by side. **b**, Illustration of ratings made by clinical, non-clinical and all graders in Test 1. The left bar denotes the true histopathological status of tested ROIs. Wirings from the left to the central bar denote ratings made in OCT step. Wirings from the central to the right bar denote ratings made in Hybrid step. Grey wirings represent correct ratings. Ratings of normal ROIs as abnormal ones are illustrated with red wirings (overdiagnosis). Ratings of abnormal ones as normal ones are illustrated with purple wirings (underdiagnosis). **c**, Illustration of ratings made by clinical, non-clinical and all graders in Test 2. The left, central and right bar denote true histopathological status of tested ROIs, ratings made in OCT step and ratings of Hybrid step, respectively. Rating a lower-grade lesion to a higher-grade lesion is illustrated by red wirings (overdiagnosis). The ratings vice versa are illustrated by purple wirings (underdiagnosis). Lesion grades increase from NSM to GM, IM, then to D, and ultimately to IMC. Source data

To assess the effectiveness of using O2E features to distinguish normal mucosa, gastric metaplasia, intestinal metaplasia, dysplasia and intramucosal cancer in BE, we recruited three expert gastroenterologists and five non-clinical scientists, blinded to the results of histopathology, as graders to classify BE mucosa based on O2E features. A flowchart-based O2E classification system (Supplementary Note 12) incorporating recurrent O2E features (Table 1) in aforementioned mucosal types was developed to aid the graders in classification. The classification tests, as illustrated in Fig. 6a, included a training phase and two formal tests. In the training phase, graders familiarized themselves with O2E features using exemplary images from 13 ROIs (1 normal, 3 gastric metaplasia, 3 intestinal metaplasia, 4 dysplasia and 2 intramucosal cancer). Then, graders were asked to classify the remaining 48 ROIs, presented in a randomized fashion, utilizing the O2E classification system. The classification tests were first performed by observing only the OCT images and subsequently by observing both OCT and OPAM images. These two-step classifications were performed for assessing the benefits of combining OCT and OPAM in classifying BE mucosa. Therefore, in each of the classification step, a total of 384 ratings (8 graders times 48 ROIs) were obtained, as illustrated in Fig. 6b,c.

**Table 1:** Description of en face and cross-sectional O2E features for classifying different BE mucosal types, mean ± s.d

Mucosal type	En face features		Cross-sectional features				
	EP inhomogeneity (a.u.)	Tumour vascular pattern	Mucosa stratification can be seen in OCT images	Glands exist within EP	Transparent glands exist in OCT images	OCT penetration (µm)	Vascular depths in OPAM (µm)
**NSM**	0.098 ± 0.02	No	Yes	No	No	726 ± 43	534 ± 114
**GM**	0.14 ± 0.04	No	No	No	No	316 ± 35	328 ± 87
**IM**	0.15 ± 0.02	No	Likely (in 75% ROIs)	Yes	No (mean transparency 69% (a.u.))	599 ± 29	264 ± 59
**D**	0.19 ± 0.08	No	Possibly (in 26% ROIs)	Yes	Yes (mean transparency 85% (a.u.))	584 ± 27	284 ± 92
**IMC**	0.14 ± 0.02	Yes	No	No	No	346 ± 24	276 ± 35

a.u., arbitrary unit; D, dysplasia; EP, epithelium; GM, gastric metaplasia; IM, intestinal metaplasia; IMC, intramucosal cancer; NSM, normal squamous mucosa.

In Test 1, we study whether en face O2E features can be used to differentiate between normal and abnormal ROIs. Therefore, in OCT step, graders were asked to classify 48 ROIs as normal or abnormal mucosa based on OCT 100–200-µm depth en face images. The results show per-ROI overall accuracy, sensitivity and specificity of 87%, 92% and 63%, respectively (Fig. 6b), with moderate interobserver agreements (Krippendorff’s *α* coefficient; Methods) among clinical (*α* = 0.41 ± 0.23, 95% confidence interval (CI)), non-clinical (*α* = 0.3 ± 0.19, 95% CI) and all (*α* = 0.36 ± 0.18, 95% CI) graders. In Hybrid step, graders reconsidered the ratings made in OCT step by reading OCT 100–200-µm depth en face images and OPAM en face images side by side. The results of Hybrid step demonstrate significantly improved accuracy, sensitivity and specificity (Fig. 6b) of 100% in distinguishing normal and abnormal ROIs with perfect interobserver agreements (*α* = 1.0).

In Test 2, graders classified each ROI into normal, gastric metaplastic, intestinal metaplastic, dysplastic or cancerous mucosa. In OCT step, OCT 100–200-µm depth en face and cross-sectional images were simultaneously assessed by graders. Results show per-ROI accuracies in identifying normal, intestinal metaplasia and dysplasia of 98%, 93% and 93% (Fig. 6c), respectively, for all graders. Misinterpretations mainly results from the difficulty in distinguishing between intestinal metaplasia and dysplasia (Fig. 6c). Overall accuracies in identifying gastric metaplasia and intramucosal cancer are good with 85% each attributed to the strikingly different features of gastric metaplasia and cancer compared with other types; thus, graders rarely misinterpret other types as gastric metaplasia or cancer (Fig. 6c). However, sensitivity is relatively poor with 65% for gastric metaplasia and 44% for cancer. Interobserver agreements in OCT step are moderate among clinical (*α* = 0.56 ± 0.13, 95% CI), non-clinical (*α* = 0.6 ± 0.09, 95% CI) and all graders (*α* = 0.59 ± 0.09, 95% CI). Categorizing dysplastic and intramucosal cancer as neoplasia, the per-ROI neoplasia detection sensitivity and specificity in OCT stage are 77% and 80%, respectively. In Hybrid step, by assessing OCT 100–200 µm depth en face, OCT cross-sectional and OPAM en face images simultaneously, graders reconsidered ratings made in the OCT step. Results show that in Hybrid step, significantly (*P* < 0.001) improved sensitivities of 96% and 88% in identifying gastric metaplasia and cancer can be achieved, respectively (Fig. 6c), and graders were also able to correct a majority (70%) of overdiagnosis of normal mucosa as intestinal metaplasia or dysplasia (Fig. 6c) made in OCT step. However, ratings of intestinal metaplasia and dysplasia were not significantly (*P* = 0.53) changed in this Hybrid step. General interobserver agreements in Hybrid step are substantial among clinical (*α* = 0.8 ± 0.07, 95% CI), non-clinical (*α* = 0.79 ± 0.07, 95% CI) and all graders (*α* = 0.8 ± 0.07, 95% CI). The overall per-ROI accuracy, sensitivity and specificity in identifying neoplasia are improved significantly from 78%, 77% and 80% in OCT step to 91%, 94% and 92% in Hybrid step, respectively. The per-patient accuracy, sensitivity and specificity are 94%, 94% and 100%, respectively.

## Discussion

BE is an acquired condition resulting from chronic oesophageal mucosal injury. As the only known precursor of EAC, BE has a relatively long time of progression to cancer^64^. Guidelines recommend screening for BE in high-risk populations and surveillance of patients with BE to identify BE neoplasia at an early stage^65^. However, based on high-definition WLE and targeted biopsies, conventional screening and surveillance are invasive and costly. Moreover, even conducted with random four-quadrant biopsies according to the Seattle Protocol, current surveillance still misses considerable inconspicuous BE neoplasia under WLE. Here we report a demonstration of a tethered capsule endoscopy combining OCT and OPAM for identifying BE neoplasia. We show that O2E resolves detailed mucosal morphology and vascular architecture of long segments of mucosa in a label-free way. Through a pilot clinical study with ex vivo resection specimens, O2E demonstrates unique and complementary features for identifying focal lesions in BE. The complete set of features allows a development of O2E classification systems to detect BE neoplasia with high accuracies.

O2E offers rich OCT and OPAM features for identifying heterogeneous mucosal types in BE. Most of the demonstrated OCT features are consistent with previous findings. The layered mucosal architecture with an intact basement membrane exhibited in this study is a well-known feature of normal squamous mucosa in OCT^66^. In mucosa of gastric types, OCT has also shown tubular pits, a total loss of mucosal stratification and low OCT penetration^27,28,67,68^ due to strong back scattering in the thick glandular epithelial compartment^67^. For intestinal metaplasia, an OCT biomarker is the partial effacement of mucosal stratification^32,35,66^. The OCT features of dysplasia were clustered and dilated glands, and a likely disappearance of mucosal stratification compared with intestinal metaplasia^32,34,35,66^. EAC was also shown in OCT as a homogeneous subsurface structure with a reduced penetration due to the high-scattering malignant cells^29^. While these OCT studies predominantly employed 1,300-nm-centre-wavelength lasers with 20–40-µm lateral resolutions and 5–10-µm axial resolutions^27,28,32,34,35,66–68^, we adopted the 1060-nm wavelength in O2E to deliver an enhanced contrast in BE imaging, owing to the stronger light-tissue interactions at the shorter wavelength^36^, and designed the optical system to generate higher lateral resolution (10 µm) mucosal imaging. Although the imaging depth was lower than 1300-nm studies, our OCT modality provides clear visualizations of microstructures, including gastric pits, epithelial crypts, intervening connective tissues in the epithelium, dilated dysplastic glands and muscle fibres. We also show OCT visualization of superficial lamina propria—a feature we found to be more common in intestinal metaplasia than dysplasia—caused by mucosal remodelling in BE. In the future, by adopting lasers with higher bandwidths and using a higher power on the tissue (Supplementary Note 5), the quality of OCT imaging can be further improved.

We implemented OPAM in endoscopy through an engineering advance of a miniaturized ultra-broadband transducer. Using the haemoglobin-sensitive 532-nm wavelength, we show that OPAM reveals delicate mucosal vasculatures. The bandwidth allows highlighted visualization of microvasculatures through high-frequency-band reconstruction. The 3D imaging ability uncovers strikingly different vascular distributions between normal and abnormal mucosae in BE. The imaged honeycomb-like vascular networks around crypts in intestinal metaplasia are comparable to rectum epithelial vasculatures^37^. The epithelial vasculatures in dysplastic mucosa present a more disrupted pattern and it was found difficult to recognize clear vascular differences between intestinal metaplasia and dysplasia in this ex vivo clinical study. A possible reason is a reduced contrast due to destructions of vasculatures during resection. This possibility is supported by many haemorrhages found in haematoxylin and eosin (H&E) and CD31 immunostained slides. Therefore, results of classification Test 2 show that OPAM has marginal impact on the classification of intestinal metaplasia and dysplasia. Future in vivo clinical studies are necessary to verify the usefulness of OPAM for identifying these two types. The dilated tumour vessels are possibly less affected in resection than the intricate epithelial capillaries in intestinal metaplasia and dysplasia, thus OPAM reveals a characteristic EC vascular pattern in cancerous areas. The imaged tumour vessels have a mean depth of ~275 µm and their morphology matches the documented intrapapillary capillary loop pattern in superficial EC^62^. With a progression to submucosal invasions, the intrapapillary capillary loop will be replaced with more irregularly shaped neo-tumour vasculatures^62^, thus OPAM may facilitate cancer staging by imaging the changing vascular features. However, this needs to be verified in further studies as specimens herein only contained intramucosal cancer with the malignancy confined within the mucosal layer. The accurate detection of early EC using vascular features demonstrated in this study indicate that other 3D vascular imaging modalities, such as OCTA, may also complement OCT to realize enhanced neoplasia detection. However, performing OCTA endoscopically has been challenging. Since motion induces false-positive signals in OCTA, OCTA is more sensitive to tissue motion than OPAM. Moreover, OCTA will notably increase the time to survey the oesophagus because it relies on dense sampling to calculate decorrelation between sequentially acquired images at a given voxel^69,70^.

Through classification tests, we demonstrate that O2E features are interpretable to clinical and non-clinical users and high accuracies can be achieved in both groups alike. With OCT alone, results show that graders identify normal, intestinal metaplastic and dysplastic mucosae with ≥93% overall accuracies. However, OCT features are not effective in differentiating intramucosal cancer from gastric metaplasia type 2, resulting in low sensitivities in recognizing these two types (intramucosal cancer: 44%; gastric metaplasia: 65%). This leads to an unimpressive per-ROI neoplasia identification of 77% sensitivity and 80% specificity, which are comparable to the reported OCT neoplasia detection performance with pooled sensitivity and specificity of 85% and 73%, respectively^39^. The ability of OPAM to decipher the tumour vascular pattern is complementary to OCT and significantly increases per-ROI neoplasia sensitivity and specificity to 91% and 94%, respectively. The corresponding per-patient sensitivity is 94% and the per-patient specificity is 100%, thus O2E has the potential to meet the American Society of Gastrointestinal Endoscopy neoplasia detection thresholds of per-patient ≥90% sensitivity and ≥80% specificity for any new method to replace random biopsy in surveillance^9^. The benefits of multi-modality are also reflected in the synergy of OCT and OPAM features for locating abnormal mucosa in en face images. With OCT alone, graders detected abnormal mucosa with 92% sensitivity and 67% specificity. The combination of OCT and OPAM significantly increases the sensitivity and specificity to 100% alike owing to the highly specific epithelial vasculatures in abnormal mucosa. Therefore, O2E supports accurate and fast location of abnormality through en face images and subsequent detailed investigation using cross-sectional images of abnormal regions to determine the actual mucosal type.

Quantitative analysis of several O2E features, including OCT penetration, OCT transparency of gland lumens and depths of vasculatures imaged in OPAM, for example, shows statistically significant differences among normal, metaplastic, dysplastic and cancerous mucosae. Using the quantified gland lumen transparency alone, an outstanding discrimination of dysplasia from intestinal metaplasia can be achieved with an AUROC of 0.92. We expect that a development of measures for automatic feature extraction and quantification of all O2E features will enable an objective identification of focal lesions in BE. The ability of O2E to detect intestinal metaplasia, dysplasia and intramucosal cancer makes it a suitable emerging tool for BE screening and surveillance. The capsule can also be disinfected for reuse to lower the cost for these operations. However, cost-effective screening technology for BE needs to be implemented in a sedation-free manner. Tethered OCT capsules^33–35,71^ with diameters (11 to 12.8 mm) comparable to the O2E capsule (12.5 mm) have been found to be well tolerated in patients not under sedation^34,35^. However, the considerably longer length of the O2E capsule (40 mm) compared with that of OCT capsules (25 mm) may render the sedation-free swallowing of the O2E capsule challenging. The longer length is attributed to the use of a long motor (length: 15 mm) and slip rings for rotating the piezoelectric transducer at high speeds. While using customized motors may reduce the capsule length, another solution is switching to new miniaturized detectors based on all-optical ultrasound detection. Two promising designs include a sub-micrometre silicon-on-insulator resonator^72^ and a Bragg grating etalon-based optical fibre^73^, achieving bandwidths of ~230 MHz and ~150 MHz, respectively. These light-weight designs, based on optical interferometry^74^, have shown high detection sensitivity, appropriate for endoscopic applications. Conversely their performance in relation to motion, when integrated within a capsule, should be tested first before opting for use in the O2E system. A third interferometry design introduced^75^, using a sound-sensitive membrane, fabricated over a silicon-photonics microring, may also be interesting for endoscopic applications. However, current implementations with bandwidths of ~38 MHz may limit the resolution over the aforementioned detectors^72,73^.

Adding multi-wavelength illumination is a critical next step for OPAM, enabling the differentiation of tissue chromophores. As a hallmark of tumour microenvironment^76^, hypoxia can be mapped by deriving haemoglobin oxygen saturation^77^ through multispectral OPAM to enhance cancer detection. Oxygen saturation can be mapped by using two or more synchronized light sources, at different visible wavelengths^78^. Since metaplastic, dysplastic and early malignant changes in BE develop within the mucosal layer, that is superficially, linear unmixing techniques could suffice for accurate computation of tissue oxygenation^79^. Collagen is another critical marker^80^ and could be resolved by employing wavelengths in the extended near-infrared range^81^. Mapping collagen can reveal cancer-associated collagen remodelling processes in BE^82^ and secretion of collagen within EC^83^. Nevertheless, due to spectral overlap in the extended near-infrared, separation of collagen from other light-absorbing molecules, such as water or lipids, may be more challenging than resolving haemoglobin in the visible range, possibly requiring a larger number of wavelengths if accurate separation from water is required^81^. The broad wavelength tuning range of optical parametric oscillators offers a straightforward solution for exploratory studies on the OPAM spectral features. However, clinical practice may require faster and more cost-effective light sources and may limit the use of an extended range of wavelengths. A good contact of the capsule with the oesophagus wall is also critical for clinical adoption of O2E imaging. Studies of OCT capsules showed that peristaltic contractions initiated by swallowing the capsule could produce a good contact between capsule and the oesophageal wall^33,34^. However, mainstream clinical application may benefit with a design that imparts adaptability to different oesophageal dimensions, possibly by employing capsules of different sizes or using a water-inflatable balloon. The use of an articulating mechanism^34^ to appose the capsule to the oesophageal wall may also be considered. As demonstrated in our study and in former publications^32,33,37^, en face visualization is a powerful tool that reveals distinctive mucosal patterns in BE. Although cross-sectional OCT features are sufficient for identifying normal, intestinal metaplastic and dysplastic mucosae, detection of cancer relies on high-quality OPAM en face images. However, in sedation-free inspections, the imaging quality could be severely affected by tissue motion^34^. Thus, a distal longitudinal scanning mechanism^84^ may be needed for O2E to obtain high-quality en face visualizations in patients not under sedation. Finally in this study, low-grade dysplasia (LGD) and high-grade dysplasia (HGD) were categorized as a dysplasia group despite the pathologist-graded dysplastic cases as LGD or HGD in the histopathological analysis. There are several reasons for this categorization. First, dysplasia progresses to cancer on a continuous scale; therefore, there is not a well-defined cut off to separate LGD and HGD^85^. The resultant dysplasia grading is known to include substantial interobserver variability^61,86^. Second, grading of LGD and HGD is often dependent on the degree of observed cytologic atypia^61^, which are not resolvable in O2E due to the resolution limit. Hence, quantitative analysis of O2E features in LGD and HGD ROIs shows no significant differences (Supplementary Note 13) between these ROIs. Third, when it comes to treatment, dysplasia grading is of insignificant clinical relevance as LGD and HGD should both receive endoscopic eradication therapy to remove dysplastic cells^61,87,88^. Fourth, since current O2E does not allow direct discrimination of cellular features, the final diagnosis will still require histological confirmation. As we have shown accurate detection of intestinal metaplasia, dysplasia and intramucosal cancer using O2E, the role of O2E is then to find these premalignant conditions and early lesions in BE screening/surveillance to guide subsequent targeted biopsy in identified patients.

In summary, we have shown that O2E is a fast, label-free, high-resolution 3D endoscopic imaging technique with the ability to offer complementary tissue information for resolving unique features in BE neoplasia. Both techniques can visualize under the surface, reaching depths of more than 1 mm, which is a depth well suited to the volumetric study of early carcinogenesis morphological and functional features. The optoacoustic method can also reach deeper, depending on the wavelength employed, potentially extending in the future the capabilities of the modality to visualize lesion infiltration of oesophageal carcinoma. We believe that O2E holds translational potential for accurate screening and surveillance of BE in a minimally invasive way.

## Methods

### O2E capsule

The tethered distal-scanning O2E capsule has a hollow shaft motor (Maxon Group) to rotate a transducer assembly for circumferential imaging. A custom-made GRIN lens (GRINTECH) was fixed inside the transducer assembly after passing through the motor shaft. The GRIN lens is static in imaging. Other components inside the transducer assembly include a 45° reflective mirror and a custom-made, spherically focused, ultra-broadband LiNO3 ultrasound transducer (Sonaxis). The hollow transducer had a 1.2-mm-diameter aperture to allow the propagation of OCT and OPAM beams. The aperture was sealed with a transparent optical glass to isolate the mirror, GRIN lens and slip ring from the coupling medium (deuterium oxide) for OPAM. The transducer features a 68.5-MHz central frequency with a 5.2-mm-diameter active element and a 6.5-mm focal length in water. The GRIN lens was assembled with a double-clad fibre (DCF; Fibercore). The capsule is encapsulated with a removable, transparent polymethyl methacrylate cap. The surface of the cap has a 4° slope to reduce back-reflection for OCT. A thin carbon fibre is fixed longitudinally at the cap surface as the marker for aligning consecutive circumferential scans. The capsule is connected to a flexible sheath (diameter 3.75 mm), which serves as a tether and encloses DCF and electric wires.

### Portable O2E system

The O2E system is composed of state-of-the-art swept-source OCT and OPAM systems (Supplementary Note 1). O2E is housed inside a portable clinical cart to enable fast imaging in an endoscopy room (Supplementary Note 1). For OCT, an all-semiconductor Vernier-tuned distributed Bragg reflector laser (Insight Photonic Solutions) with a central wavelength of 1,060 nm, a top-hat spectrum bandwidth of 70 nm and an output power of 65 mW is used as the laser source. The laser has an 85-kHz sweep rate and a 400-MHz wavelength transition rate. A dual-balanced Mach-Zehnder interferometer consisting of a 90/10 fibre coupler (TW1064R2A2A, Thorlabs) and a 50/50 (TW1064R5A2A, Thorlabs) fibre coupler generates OCT interference fringes. A balanced photodetector (Insight Photonic Solutions) transfers the fringes into electrical signals for data acquisition (DAQ).

For OPAM, a pulsed 532-nm Q-switched laser (Onda 532 nm, Bright Solutions) with a pulse width of 2 ns is used as the light source. The incident pulse energy on the sample is 12 µJ. The OPAM beam is combined with the OCT beam through a double-clad fibre coupler (DCFC; DC1060LEFA, Thorlabs). OPAM and OCT light is coupled into the multimode and single-mode input ports of the DCFC, respectively. The output port of DCFC is fusion spliced with a DCF to transfer the light into the capsule. A transistor–transistor logic frequency divider (PRL-220A, Pulse Research Lab) divides the OCT sweep trigger by half to trigger OPAM laser at half the rate of OCT laser. As a result, axial scan (A-scan) rates of OCT and OPAM are 85 kHz and 42.5 kHz, respectively. The OPAM signal from the transducer is amplified 30 dB with a custom-made preamplifier (Sonaxis). The amplified signal passes through a T-bias (ZFBT-4R2GW+, Mini-Circuits) to a DAQ device.

Synchronous acquisition of OCT and OPAM signals is realized using a dual-channel DAQ device (ATS9373, AlazarTech). The OCT laser provides a 400-MHz master clock timed with the wavelength transitions inside the laser cavity. The DAQ adopts the 400-MHz master clock as the sample clock and acquires dual-modal data in continuous mode. A home-built Microsoft Windows application (RayFos) integrates DAQ with real-time image visualization.

### Characterization of O2E imaging

To characterize O2E imaging, the capsule was mounted on 2D motorized stages (M511.DD1, Physik Instrument). For OPAM, the Fourier-transformed A-scan of a 7-µm-diameter carbon fibre reveals a broadband detection with a central frequency of 68.5 MHz and a −6-dB bandwidth of 100 MHz (Supplementary Fig. 1c). Utilizing the full bandwidth (3–110 MHz) in the optoacoustic signal, the Hilbert transformed A-scan shows a full-width at half-maximum (FWHM) of 17 μm (Supplementary Fig. 1d), which is the axial resolution of OPAM. The edge of a resolution target (3″ × 3″ Positive, USAF 1951, Edmund Optics) was raster scanned (2.5-µm X-axis step size for OPAM, 1-µm X-axis step size for OCT) in water to measure OPAM and OCT lateral resolutions. Through maximum intensity projection for OPAM and average intensity projection for OCT, edge-spread functions were obtained and negative derivatives of edge-spread functions were fitted using Gaussian models to obtain line spread functions for the two modalities. Corresponding FWHMs of the line spread functions show 30-µm lateral resolution for OPAM (Supplementary Fig. 1d) and 10-µm lateral resolution for OCT (Supplementary Fig. 1i). A 160-µm OPAM depth of field (−6 dB) was measured (Supplementary Fig. 1e) by raster scanning the carbon fibre in the Z axis (2.5-µm step size). The axial resolution of OCT was measured as 9.75 μm in air by taking the FWHM of OCT A-scans of a mirror surface (Supplementary Fig. 1j). The corresponding axial resolution in tissue is 7 μm, assuming a tissue optical refractive index of 1.38. The signal-to-noise ratio of OCT is 102 dB in water with a −6-dB depth of field of 600 μm (Supplementary Fig. 1k). The OCT signal-to-noise ratio was measured with 5-mW incident power on the mirror surface and 30-dB optical attenuators in the sample arm. Data processing was implemented in MATLAB (MathWorks).

### OPAM and OCT image registration

A precise registration of the two modalities was achieved for O2E imaging. The integration of OPAM and OCT light through a DCFC and a GRIN lens produced inherently co-axial dual-modal illumination at samples. The optical focus of OCT and the acoustic focus of OPAM were aligned in the process of capsule assembling. In addition, helical scanning of a metal mesh phantom (Supplementary Note 2) was implemented to register OPAM and OCT images. OPAM data was first interpolated (nearest-neighbour interpolation) to achieve identical pixel sizes as OCT. An optimal axial shift number was then searched to align OPAM and OCT in the axial direction. Supplementary Note 2 shows precisely co-registered OPAM and OCT images following the above steps. The processing was implemented in MATLAB.

### En face OPAM and OCT images

En face images of OPAM and OCT were generated by maximum intensity and average intensity depth projection within a desired depth range of 3D OPAM and OCT data, respectively.

### OPAM reconstruction using different frequency bands

To sustain a high imaging speed, tissues were not redundantly sampled by the transducer. Therefore, OPAM image reconstruction was performed by frequency filtering a time-domain optoacoustic signal according to the desired frequency band, Hilbert transforming the signal and utilizing the processed signal as an A-scan. For the full-bandwidth reconstructed image, the frequency band of 3 to 110 MHz was used. The low-frequency band and high-frequency band images were reconstructed by utilizing 3 to 40-MHz components and 40 to 110-MHz components in the optoacoustic signals, respectively. Components higher than 110 MHz were discarded due to increased noise.

### Clinical pilot study with in vivo imaging in a healthy volunteer

An ethic waiver (2022-495-W-KH) was granted by the Ethics Commission of the Technical University of Munich for imaging the oral mucosa of a healthy volunteer (male, 30 years old) using O2E. Before imaging, informed consent was obtained from the volunteer. To obtain the image, the volunteer put on safety goggles and wrapped the labial mucosa around the capsule. A motorized helical scan was performed (2.4-mm s^−1^ pullback speed, 20-mm pullback distance, 30-Hz rotational speed) with 12-µJ pulse energy for OPAM and 5-mW incident power for OCT. Imaging process took 8.5 s.

### Ex vivo and in vivo swine imaging

An ethics protocol (ROB-55.2-2532.Vet_02-18-33) was approved and granted by the Ethics Committee of the responsible authority (Regierung von Oberbayern) for imaging swine oesophagus ex vivo and in vivo using O2E. For ex vivo imaging, a segment of pig oesophagus (Supplementary Fig. 4a) was obtained from a sacrificed crossbred swine (German Landrace with minipig) weighing 27 kg. The oesophagus was pinned inside a tube. Black surgical sutures (100-µm diameters) were embedded inside the oesophagus as artificial optical absorbers for OPAM. Imaging was performed through motorized pullback (2.4 mm s^−1^, 10-mm pullback distance, 30-Hz rotational speed). For in vivo imaging, a 6-month-old, 60-kg crossbred swine (German Landrace with minipig) was anesthetized using a combination of ketamin (0.2 ml kg^−1^) and azaperon (0.05 ml kg^−1^). The anesthetized animal was laid on an elevation table (Supplementary Fig. 5a) and a hollow mouth guard was inserted to keep its mouth open (Supplementary Fig. 5b). The O2E capsule was inserted through the mouth guard into its oesophagus. Insertion stopped when the capsule was estimated to reach the lower oesophagus. The capsule was pulled back manually, and in vivo oesophageal imaging was conducted at 30-Hz rotational speed. After imaging, the swine recovered after being intramuscularly administered with meloxicam (0.5 mg kg^−1^). For swine imaging, a pulse energy of 12 µJ was applied for OPAM and the incident power on the sample for OCT was 5 mW.

### Patient recruitment

An ethics protocol (18/EM/0069) was approved and granted by East Midlands—Nottingham 2, Research Ethics Committee of The National Health Service to recruit patients with BE and conduct ex vivo study on resected tissue specimens. A total of 14 patients suspected of BE neoplasia agreed to be recruited at Cambridge University Hospital for this study. Consent forms were obtained from these patients. Experienced clinicians examined the patients endoscopically. EMR was performed on 10 patients who were found suitable for resection. Characteristics of patients receiving EMR can be found in Supplementary Note 6.

### Clinical pilot study with imaging of ex vivo EMR specimens

Endoscopic resection specimens were pinned on silicon supports with 20-mm Agani needles. The silicon support was mounted on a custom-made specimen holder (Supplementary Note 7), which allowed the uniform concave deformation of the support and the clinical specimen to be maintained. The pinned specimens were imaged in an endoscopy room within 30 min after resection. O2E imaging was realized by a motorized pullback at 1-mm s^−1^ pullback speed and 20-Hz rotational speed. During imaging, the capsule was placed in light contact with the specimen to mimic luminal oesophageal imaging. Average imaging time was 40 s for a specimen. A pulse energy of 12 µJ was illuminated on the mucosa for OPAM imaging. The OCT incident power was 5 mW.

### Histopathology of EMR specimens

After fixation, EMR specimens were stained on the lateral margin to maintain the orientation. The specimens were sectioned every 2 mm along the horizontal axis with the first section corresponding to the 12 o’clock position (top) and the last to the 6 o’clock position (bottom). The sections were sliced, mounted on glass slides and stained with standard H&E. To ensure high-quality H&E slides, each section was sliced three times with an inter-slice distance of ~100 µm. Obtained H&E slides were scanned with a slide scanner microscope (Axioscan). An expert gastrointestinal pathologist assessed the pathology slides on a millimetre-per-millimetre basis from 9 to 3 o’clock on digital files (.zen format) with glass slides as reference. Each millimetre grid cell was classified into one of the following mucosal types: normal squamous mucosa, gastric metaplasia, intestinal metaplasia, dysplasia or intramucosal cancer. In this process, the pathologist made note of whether there was artefact (damaged epithelium) or overlay of squamous over columnar epithelium. Based on the pathology reporting, pathology grids were generated excluding regions with artefacts. The pathology grids were superimposed over the bright field images of the specimens. To inform the best and most plausible correlation between pathology grids and bright field images, reference elements (for example, shape of the specimens, metal pin and macroscopic evidence of colour difference indicating different epithelium) were taken into account. Based on the pathology grids, each specimen was divided into separate regions of different mucosal types. A total number of 61 regions with different sizes were identified from all 14 EMR specimens.

### CD31 immunostaining

Immunohistochemistry was performed on a total of 17 ex vivo sections using the Bond RXm system (Leica) and primary antibody against CD31 (ab28364; Abcam) diluted 1:50 in Bond Primary Antibody Diluent (AR9352, Leica). Slides were deparaffinized using the BOND Dewax Solution and treated for 20 min with Epitope Retrieval Solution 1 and heat (100 °C). Antibody binding was detected with a Bond Polymer Refine Detection kit (DS9800, Leica) without post-primary reagent and visualized with DAB as a dark brown precipitate. Immunostained slides were counterstained with haematoxylin. Correlation of CD31 slides with O2E can be found in Supplementary Notes 8 and 9.

### Quantification of en face O2E features

En face features of O2E, including epithelial homogeneity and vascular density, were quantified in all ROIs. The epithelial homogeneity was measured based on OCT 100–200-µm depth-projection en face images. MATLAB function roipoly was used to manually define the ROI in the OCT 100–200-µm en face image. The ROI was then divided into 2 × 3 mm^2^ rectangular grids (Supplementary Note 11) and inside each grid cell a coefficient of variation *C*_v_ was computed as:

$${C}_{{\mathrm{v}}}=\frac{\sigma }{\mu }$$

where *μ* and *σ* are the mean pixel value and standard deviation of pixel values, respectively. The mean value of *C*_v_ values of all grid cells inside the ROI was retrieved as the ROI epithelial inhomogeneity. Vascular density was quantified using high-frequency band OPAM data. After Hilbert transforming OPAM A-scans within a selected ROI, a thresholding was applied to select valid A-scans with maximum signal amplitudes of at least five times the noise floor level. The noise floor was defined as the variance of the background noise. The proportion of valid A-scans to all A-scans of an ROI was retrieved as its vascular density. Data processing was implemented in MATLAB.

### Quantification of cross-sectional O2E features

Cross-sectional features include OCT penetration depth, gland transparency and depths of blood vessels. OCT penetration depth was quantified in all ROIs, while gland transparency was only quantified in intestinal metaplastic and dysplastic ROIs. OCT cross-sectional images were averaged over five consecutive ones to reduce the noise for quantifying the OCT penetration. A thresholding was implemented to select valid OCT A-scans with a maximum signal amplitude of at least three times the noise floor level. The noise floor corresponds to the background variance. The penetration depth of each A-scan was calculated as the depth of the deepest pixel with a value of at least three times of the noise floor level. The mean value of the depths of all valid OCT A-scans within an ROI was retrieved as its average OCT penetration depth. OCT transparency of the glands was measured in OCT cross-sectional images. Pixel values inside gland lumens were manually extracted using MATLAB function roipoly. Glands that appeared at the same location in consecutive cross-sectional images were regarded as the same gland. The OCT transparency of a gland was quantified as:

$${{\mathrm{Transparency}}}=\left(1-\frac{{{{{\mathrm{Mean}}\;{\mathrm{value}}\;{\mathrm{of}}\;{\mathrm{all}}\;{\mathrm{pixels}}\;{\mathrm{within}}\;{\mathrm{a}}\;{\mathrm{gland}}}}}}{{{{{\mathrm{Numerical}}\;{\mathrm{upper}}\;{\mathrm{limit}}\;{\mathrm{of}}\;{\mathrm{pixel}}\;{\mathrm{values}}}}}}\right)\times 100 \%$$

Therefore, a 100% OCT transparent gland means its interior does not reflect any OCT light to the detector. Vascular depth was quantified using high-frequency band OPAM data. After Hilbert transforming OPAM A-scans within a selected ROI, a thresholding was applied to select valid A-scans with maximum signal amplitudes of at least five times the noise floor level. The noise floor was defined as the variance of the background noise. The mean value of the axial locations of the maximum pixel in each valid A-scan was retrieved as the average depth of blood vessels within the corresponding ROI. Data processing was implemented in MATLAB.

### O2E classification system and its validation

En face and cross-sectional O2E features were used to develop a flowchart-based O2E classification system (Supplementary Note 12) to guide graders in classifying BE mucosa. The classification system consists of two flowchart algorithms. The first flowchart was designed to guide the user to identify abnormal regions in classification Test 1 using en face images. The second flowchart was developed to guide the user to classify an ROI into normal squamous mucosa, gastric metaplasia, intestinal metaplasia, dysplasia or intramucosal cancer in classification Test 2 using en face and cross-sectional features. Graders were trained to follow the flowchart algorithms for the tests. During tests, 48 ROIs were randomized and O2E images from each ROI were organized as Microsoft PowerPoint presentations for graders. Graders recorded their ratings in Microsoft Excel spreadsheets. An investigator (Q.L.) collected the rating results afterwards. There was no time limit for training and classification tests. Graders completed the training and tests within an average of 3 h.

### Statistical analysis

Descriptive statistics were recorded as quartiles in Box and whisker plots. The significance of differences between samples were estimated using the two-sided Wilcoxon rank sum test in MATLAB. The empirical receiver operating characteristic (ROC) curve was generated in MATLAB by connecting the discrete pairs of true positive rates and false-positive rates. The nonparametric estimate of area under ROC was the summation of the areas of the trapezoids formed by connecting the points on the ROC curve in MATLAB. R package krippendorffsalpha was employed to measure the interrater agreement using Krippendorff’s α coefficient in R v.4.2.3.

### Statistics and reproducibility

As mentioned in histopathology of EMR specimens, to ensure high-quality H&E slides were obtained, three separate slices were taken at an interval of ~100 µm in each section. These repeated slices show very similar histopathology, although some slices can miss a certain tissue region due to damage in the preparation process. O2E imaging features can be repeatedly corresponded with histopathology in these independent slices. Therefore, correlation of O2E cross-sectional images with H&E slides (such as the representative images shown in Figs. 2g–j, 3a and Fig. 5h and Supplementary Figs. 7–9) in this study can be repeatedly verified.

### Laser safety

An incident power of 5 mW and a pulse energy of 12 µJ on the sample was adopted for OCT and OPAM imaging in this study, respectively. According to the American National Standard for the Safe Use of Lasers, the maximum permissible exposures for OCT and OPAM in this case were 257 mJ cm^−2^ and 1.93 mJ cm^−2^ (Supplementary Note 14), respectively. The actual radiant exposures applied for O2E imaging were 2.7 mJ cm^−2^ for OCT and 0.22 mJ cm^−2^ for OPAM (Supplementary Note 14). Therefore, O2E imaging did not exceed the laser safety limit.

### Reporting summary

Further information on research design is available in the Nature Portfolio Reporting Summary linked to this article.

## Supplementary information

Supplementary Information Supplementary Contents, Figs. 1–14 and References.

Reporting Summary

## Source data

Source Data Fig. 3 Statistical source data (OCT transparency of intestinal metaplastic glands and dysplastic glands, distribution of glands transparency in ROIs, OCT penetration in different types of mucosae and average vascular depth in different types of mucosae).

Source Data Fig. 4 Statistical source data (epithelial inhomogeneity in different types of mucosae, vascular density in different types of mucosae, distribution of mucosal inhomogeneity and vascular depths in different types of mucosae).

Source Data Fig. 6 Statistical source data (all ratings the 8 graders made in the validation of O2E imaging features to differentiate various types of mucosae).

## Data Availability

O2E patient data is too large to be publicly shared but is available for research purposes from the corresponding authors on reasonable request. O2E data of healthy human labial mucosa is available via Zenodo at 10.5281/zenodo.10817674 (ref. ^89^). Source data are provided with this paper.

## Code availability

O2E data were acquired via a custom-built Microsoft Windows application. This application is licensed and cannot be shared. Offline OCT reconstruction was implemented via custom-built LabVIEW code (LabVIEW 2016), which can be provided for research purposes from the corresponding authors on reasonable request. OPAM reconstruction and O2E data analysis were implemented via custom-built MATLAB code (MATLAB 2018a). The MATLAB code is available via Zenodo at 10.5281/zenodo.10817674 (ref. ^89^).
