# Supplementary material for: Tethered optoacoustic and optical coherence tomography capsule endoscopy for label-free assessment of Barrett’s oesophageal neoplasia
Source: Nat Biomed Eng. 2025 Aug 6;10(2):259–76. doi: 10.1038/s41551-025-01462-0 (PMC12920095; doi:10.1038/s41551-025-01462-0)
Supplement: Supplementary file 1 — Supplementary Contents, Figs. 1–14 and References. [file 41551_2025_1462_MOESM1_ESM.pdf]

nature biomedical engineering

Supplementary information https://doi.org/10.1038/s41551-025-01462-0

Tethered optoacoustic and optical coherence tomography capsule endoscopy for label-free assessment of Barrett's oesophageal neoplasia

In the format provided by the authors and unedited

Contents

1. O2E imaging system and its characterization 2
2. OPAM and OCT image registration 4
3. OPAM images at different frequency bands 6
4. Ex vivo imaging of swine esophagus 8
5. *In vivo* imaging of swine esophagus 9
6. Patient characteristics 11
7. EMR specimen imaging using O2E 12
8. Correlating OPAM with CD31 immunostaining 13
9. Analysis of vessel diameter and orientation through cross-sectional images 15
10. High-frequency band OPAM images of EMR specimens 17
11. Quantification of mucosal inhomogeneity 20
12. Development of O2E classification system and its validation 21
13. Quantification of O2E features in GM type 1, GM type 2, LGD and HGD 23
14. Laser safety limit 25

References 27

1. O2E imaging system and its characterization

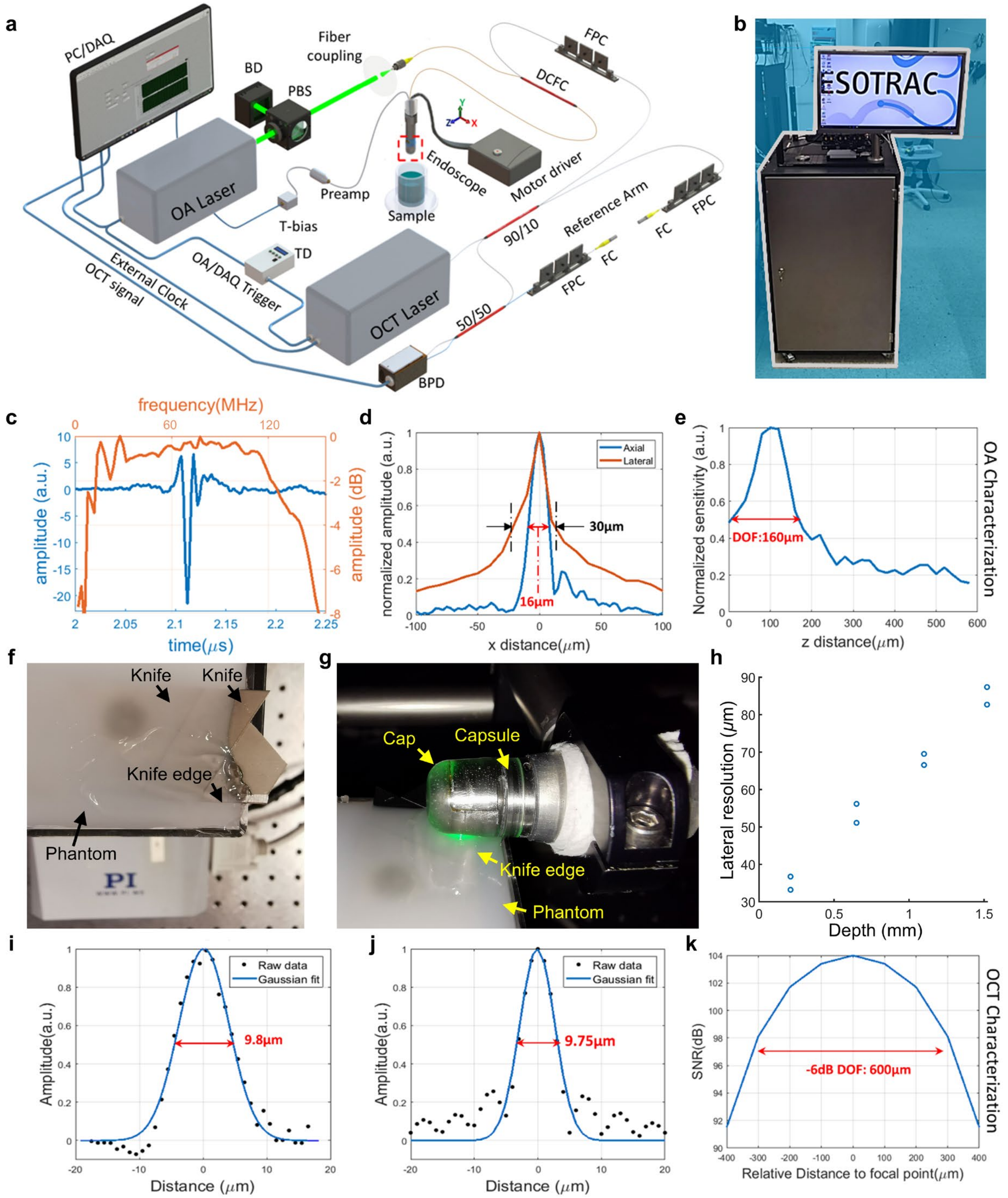

Supplementary Fig. 1| O2E imaging system and characterizations. a, A schematic diagram of O2E imaging system. OA: optoacoustic, PBS: polarizing beam splitter, BD: beam dump, DCFC: double-clad fiber coupler, FPC: fiber polarization controller, FC: fiber collimator, TD: Trigger divider, BPD: Balanced photodetector. **b**. A photo of the O2E imaging system housed inside a clinical portable cart. **c-e**, OPAM characterizations including the temporal OPAM A-scan and its Fourier transformation (**c**), axial and lateral resolutions of full bandwidth OPAM (**d**), and -6 dB depth of focus (**e**). **f**. Photo of a knife obliquely inserted into an agar

phantom added with intralipid to estimate the degradation of OPAM lateral resolution as a function of depth in biological tissues. The knife edge at different depths within the phantom was raster scanned using O2E capsule mounted on 2D mechanical stages. **g**. Photo of the O2E capsule ready for scanning the knife edge. **h**. Depth dependent OPAM lateral resolutions estimated from edge spread functions by raster scanning knife edges at different depths. **i**-**k**, OCT characterizations including the lateral (**i**) and axial (**j**) resolutions, and -6 dB depth of focus (**k**).

Suppl Fig. 1a provides a schematic diagram of the O2E imaging system. The system houses state-of-the-art swept-source OCT and OPAM systems in a portable clinical cart (**Suppl Fig. 1b**). The excitation light of the two systems is integrated through a double-clad fiber coupler (DCFC) into a tethered capsule endoscope for imaging. To characterize the imaging performances of the O2E system, various phantoms were raster scanned by mounting the capsule on motorized translation stages. To measure the detection bandwidth of the ultra-broadband OPAM detector, the broadband signal from tiny carbon fibers (7-µm diameter) was collected with the O2E capsule in water. The Fourier transformed A-scan signal (**Suppl Fig. 1c**) demonstrates a broadband detection with a -6 dB bandwidth of 100 MHz. The Hilbert transformed A-scan signal (**Suppl Fig. 1d**) shows a half-maximum (FWHM) of 16 µm, which corresponds to the axial resolution of OPAM. To characterize the lateral imaging resolution of OPAM, the edge of a resolution target (3" x 3" Positive, USAF 1951, Edmund Optics, USA) was raster scanned (2.5 µm X-axis step size) using the capsule in water. The OPAM edge-spread function (ESF) was obtained through maximum intensity projection, and its negative derivative was fitted using the Gaussian model to obtain the lateral line spread function (LSF, **Suppl Fig. 1d**). The FWHM of the LSF demonstrates a lateral resolution of 30 µm for OPAM (**Suppl Fig. 1d**). By raster scanning the carbon fiber in Z-axis (2.5 µm step size), the maximum signal intensities show a -6 dB depth of field (DOF) of 150 µm for OPAM (**Suppl Fig. 1e**). In order to characterize the possible degradation of OPAM lateral resolution as a function of depth, the sharp edge of a knife obliquely inserted into a tissue-mimicking phantom was raster scanned (**Suppl Fig. 1f & g**). OPAM ESFs were obtained at a depth range of 0.2 – 1.5 mm inside the phantom. Negative derivatives of these ESFs were fitted using the Gaussian model to obtain corresponding LSFs. By calculating the FWHM of these LSFs, it is found that the OPAM lateral resolution degrades from ~ 33 µm at 200 µm depth in the phantom to ~ 85 µm at the depth of 1.5 mm (**Suppl Fig. 1h**), due to the defocus of the transducer. A more complicated image reconstruction algorithm[1] may alleviate the resolution degradation. The phantom was prepared by adding intralipid as optical scatterers in the heated agar/water solution (1% agar). Afterwards, the knife was obliquely inserted into the solution before it solidified. With an intralipid concentration of 1% and agar concentration of 1%, the solidified phantom is homogeneous and would have a reduced scattering coefficient of ~ 1 mm^{-1} according to the literature[2], close to that of esophageal mucosa at 532 nm wavelength[3]. The OPAM performances were characterized by utilizing the full-bandwidth (3 to 110 MHz) in the optoacoustic signal. Components higher than 110 MHz were discarded due to increased noise.

Similar procedures were taken to characterize the performances of OCT modality. The edge of the resolution target was raster scanned (1 µm X-axis step size) with the capsule in water. The OCT ESF was obtained through average intensity projection. The FWHM of LSF, which was obtained by Gaussian fitting the negative derivative of ESF, shows an OCT lateral resolution of 9.8 µm (**Suppl Fig. 1i**). To measure the axial resolution of OCT, OCT A-scans of a silver mirror in air were obtained. Gaussian fitting of the A-scan shows an axial resolution of 9.75 µm through FWHM (**Suppl Fig. 1j**). The corresponding axial resolution in tissue was 7 µm, assuming a tissue optical refractive index of 1.38. The sensitivity of OCT was measured using the same mirror surface by adding 30-dB optical attenuators in the sample arm. By moving the capsule in the Z direction, the A-scans from the mirror surface shows a -6 dB DOF of 600 µm for OCT (**Suppl Fig. 1k**).

2. OPAM and OCT image registration

Although O2E produces co-axial dual-modal illuminations, additional processing is needed to co-register the two imaging modalities. A metal mesh phantom (**Suppl Fig. 2a**) was used for developing these methods. Helical scans of the phantom were implemented by mounting the capsule on XZ motorized stages (M511.DD1, Physik Instrument, Germany). The image registration steps are listed in **Suppl Fig. 2b**. As the A-scan rate of OPAM is half of that of OCT, the original inter-pixel distance of OPAM in the circumferential direction is twice the distance of OCT. In the axial direction, the original inter-pixel distances of OPAM and OCT were measured as 3.75 μm and 2.5 μm in water, respectively. Therefore, a nearest-neighbor interpolation was performed on OPAM data to achieve same inter-pixel distances as OCT. A nice lateral registration between the two modalities can be observed from full-depth projection OCT and OPAM *en face* images (**Suppl Fig. 2c**) after interpolation.

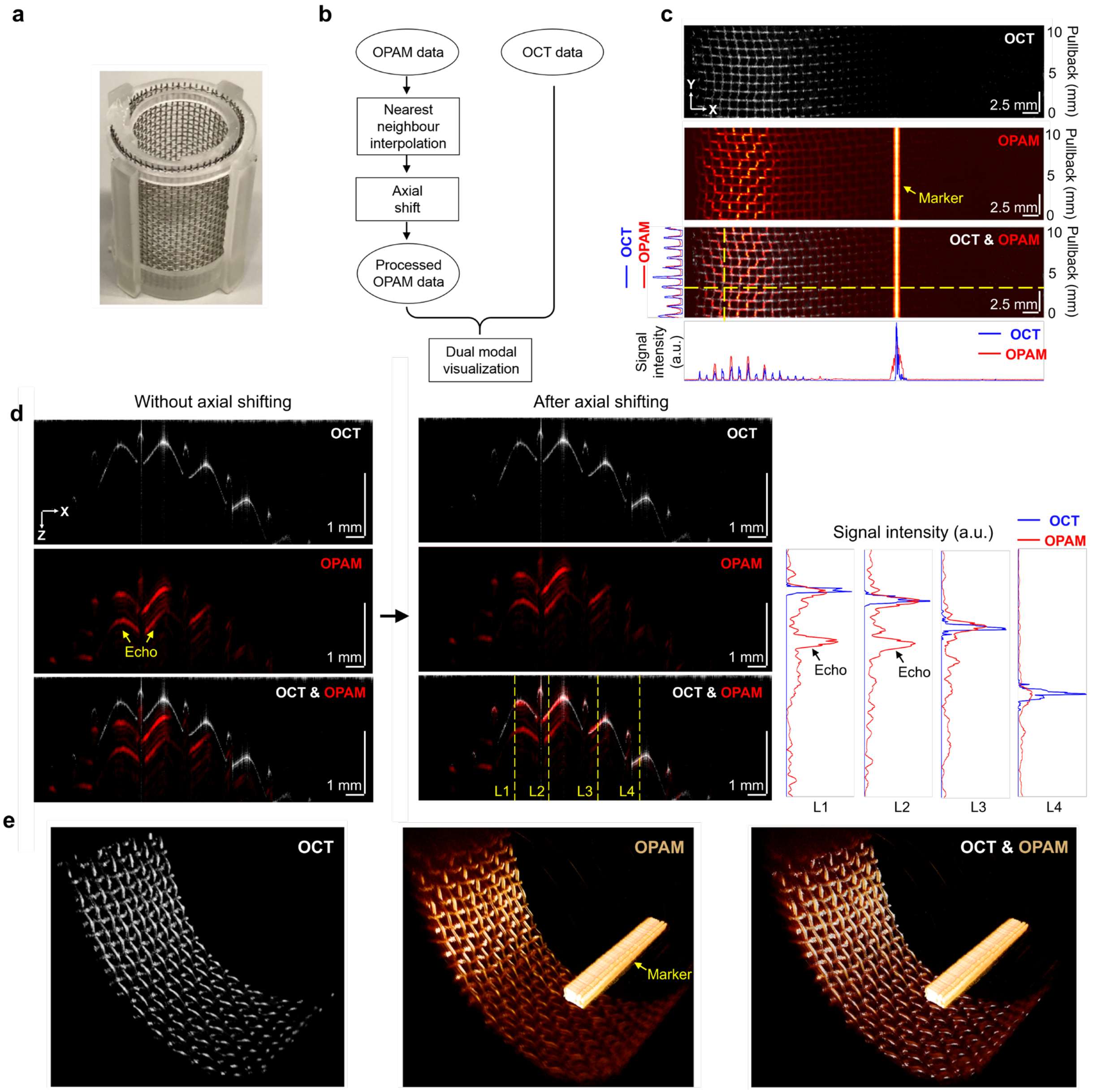

Supplementary Fig. 2| OPAM and OCT image registration. a, Photo of the metal mesh phantom for developing registration methods. **b,** Flowchart illustrating the processing steps to register the dual modal data. **c,** Full-depth projection OCT, OPAM and hybrid *en face* images of the phantom in Cartesian coordinates. The images show a nice lateral registration after interpolation of OPAM data. **d,** Representative cross-sectional OCT, OPAM and hybrid images of the phantom in Cartesian coordinates. Images show a nice alignment of the two modalities in the axial direction after axial shifting OPAM data. **e,** Three-dimensional visualization of registered OCT, OPAM and hybrid imaging of the phantom.

The misalignment in the axial direction (**Suppl Fig. 2d**) was corrected by shifting OPAM data in this direction. An optimal axial shift number was searched through an iteration of shift numbers for OPAM. The axial shift producing maximum cross-correlation between OCT and OPAM cross-sectional images was obtained as the optimal one. This optimal shift number was then used for correction of all OPAM images in this study. Axial shifting was able to produce a satisfactory alignment between the two modalities (**Suppl Fig. 2d**). **Suppl Fig. 2e** shows registered OPAM and OCT imaging of the mesh phantom in 3D.

3. OPAM images at different frequency bands

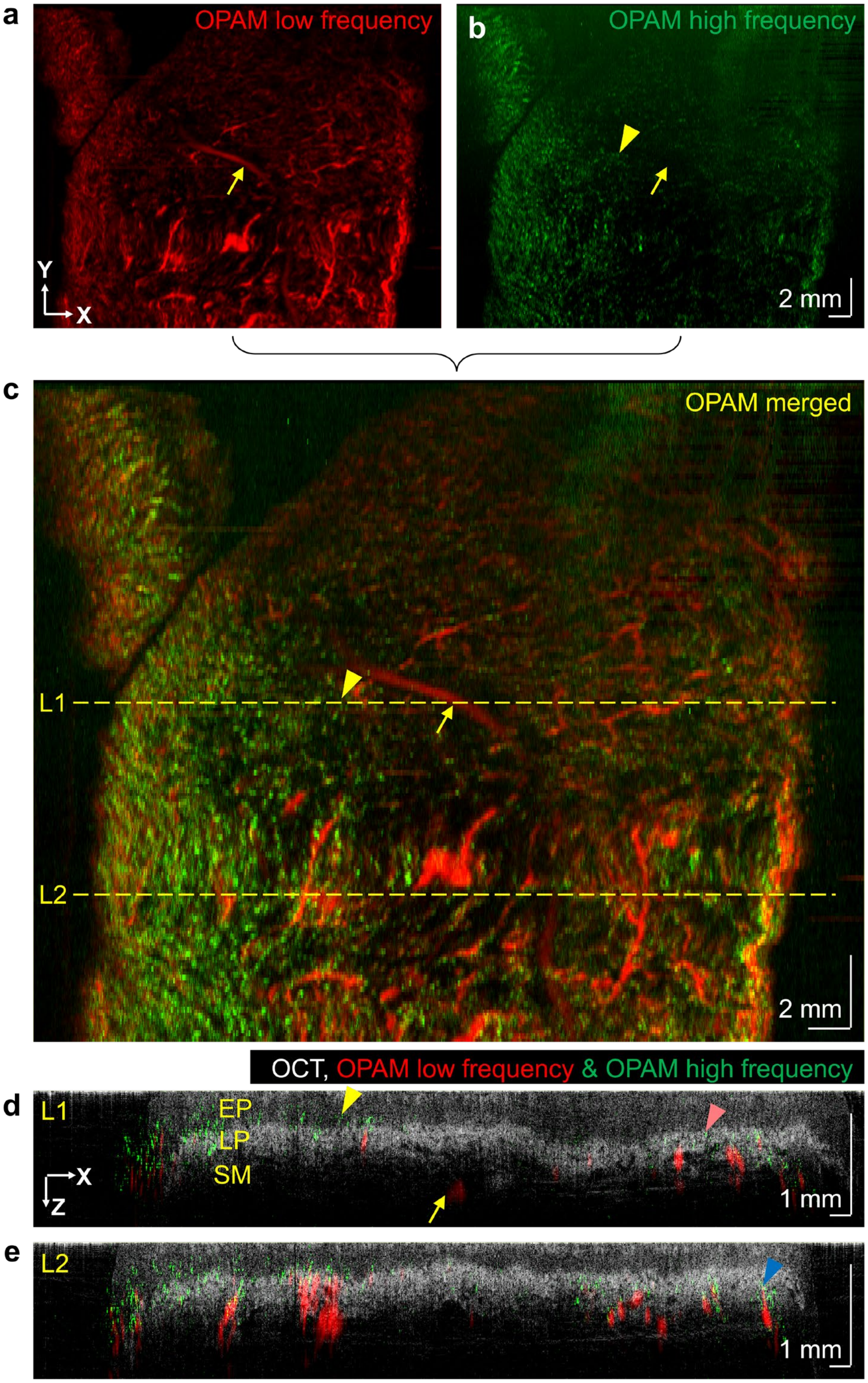

Supplementary Fig. 3| OPAM images at different frequency bands. a-b, Full-depth projection *en face* OPAM images of the human labial mucosa reconstructed at the low frequency band (3 – 40 MHz) and high frequency band (40 – 110 MHz), respectively. **c**. The *en face* OPAM image of the labial mucosa by merging the low frequency band and high frequency band images. **d-e,** Merged cross-sectional images of the labial mucosa at locations of dashed yellow lines in **c**. Yellow arrow: a large blood vessel that is visible in the low-

frequency band OPAM, but invisible in the high-frequency band OPAM. Yellow arrowhead: capillary loops inside papillae. Pink arrowhead: small vasculatures in the lamina propria. Blue arrowhead: the high-frequency component generated from large blood vessels. EP, epithelium. LP, lamina propria. SM, submucosa.

The ultra-broadband detection allows OPAM to highlight vascular features of different sizes by using different frequency bands to reconstruct images. **Suppl Fig. 3a-b** compares the *en face* low-frequency band (3 – 40 MHz) and high-frequency band (40 – 110 MHz) OPAM images of human labial mucosa. The two images show markedly different vascular features. The low-frequency image specifically shows the large vascular network in lamina propria and submucosa (**Suppl Fig. 3a**). The high-frequency image mainly shows papillary capillary loops appearing as round dots (**Suppl Fig. 3b)** due to the papillae extending normally into the epithelium. Large blood vessels visible in the low-frequency image (e.g., the blood vessel pointed with the yellow arrow) are missing in the high-frequency image. By merging the images of different frequency bands, **Suppl Fig. 3c** provides an enhanced visualization of the high frequency components, which could be masked by the strong low-frequency components without frequency banding. Merged cross-sectional images combined with OCT (**Suppl Fig. 3d-e**) shows that the high-frequency components correspond to high-frequency signals generated from capillary loops in the papillae (e.g., yellow arrowhead) and small blood vessels in lamina propria (e.g., pink arrowhead), as well as the high-frequency part of large blood vessels (e.g., blue arrowhead).

4. Ex vivo imaging of swine esophagus

A segment of healthy swine esophagus (**Suppl Fig. 4a**) was obtained for *ex vivo* O2E imaging test. The esophagus was pinned inside a tube. As the mucosa was drained of blood, black surgical sutures were embedded inside the esophageal wall as optical absorbers for OPAM. The esophagus was then filled with water. O2E capsule was inserted into the esophagus and a motorized 15-mm pullback of the capsule fulfilled 3D esophageal imaging (**Suppl Fig. 4b**). From the cross-sectional images (**Suppl Fig. 4c-e**), OCT clearly shows a layered mucosal architecture with a squamous epithelium, high scattering lamina propria and the muscularis mucosae layer beyond the lamina propria. Glandular structures can be identified beneath the epithelium. As almost all blood had drained, OPAM only shows the embedded sutures (**Suppl Fig. 4f-h**) and the marker for aligning consecutive cross-sectional images. Therefore, this *ex vivo* imaging serves as a negative contrast demonstrating that at 532 nm wavelength, blood is the major prominent optical absorber in the esophageal mucosa.

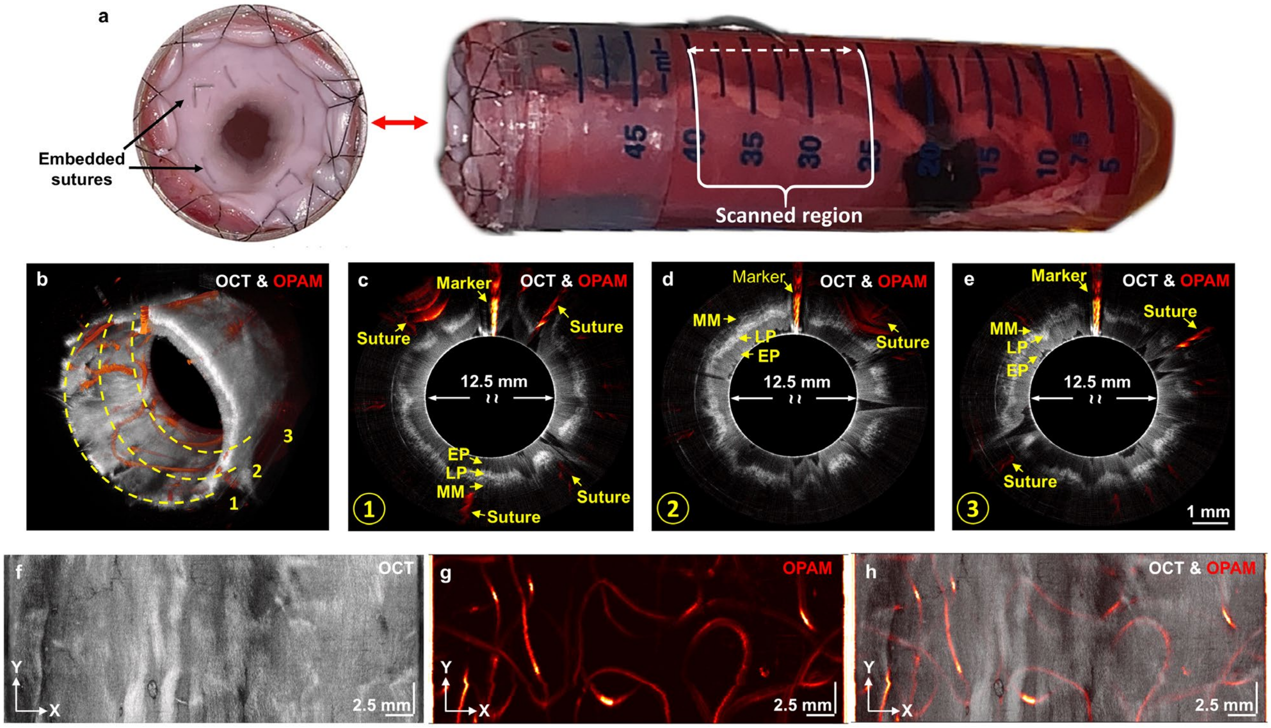

Supplementary Fig. 4| *Ex vivo* swine esophageal imaging. a, A photo of a segment of swine esophagus pinned inside a tube for O2E imaging. Black sutures were embedded inside the esophageal wall. **b,** Three-dimensional rendered O2E imaging of the *ex vivo* swine esophagus. **c-d,** Cross-sectional hybrid images of the swine esophagus corresponding to yellow dashed lines in **b**. EP, epithelium. LP, lamina propria. MM, muscularis mucosae. **f-h**, Full-depth projection OCT, OPAM and hybrid *en face* images of the *ex vivo* swine esophagus in Cartesian coordinates, respectively.

5. *In vivo* imaging of swine esophagus

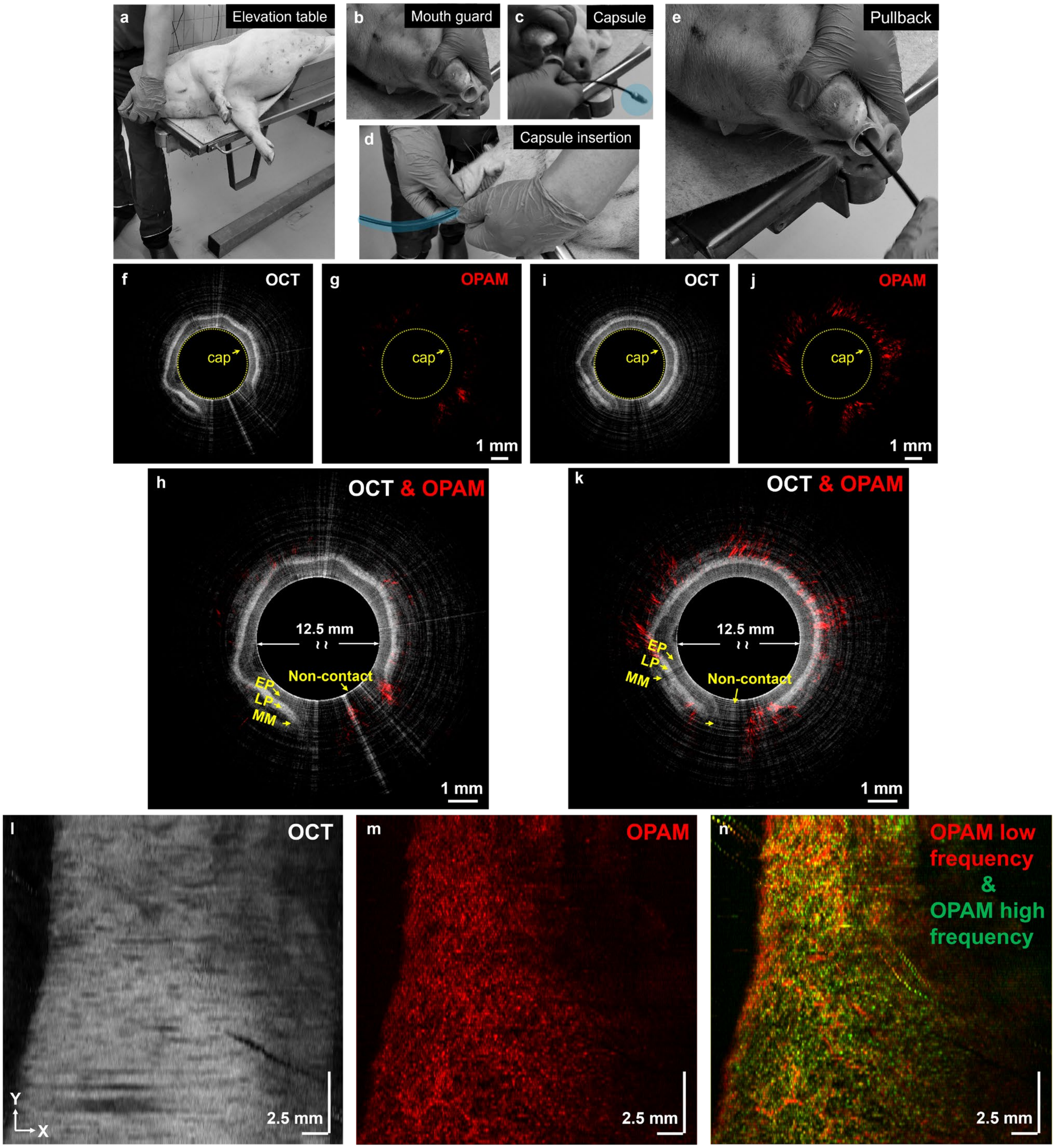

Supplementary Fig. 5| *In vivo* imaging of swine esophagus. a-e, Steps of in vivo swine imaging, including placing the anesthetized animal on an elevation table (**a**), inserting a hollow mouth guard between the teeth to keep the mouth open (**b**), inserting the O2E capsule into the distal esophagus through the mouth guard (**c-d**), and manually pulling back the capsule to helically scan the esophagus (e). **f-h,** Representative cross-sectional OCT (**f**), OPAM (**g**) and hybrid (**h**) images of the healthy esophagus show layered mucosal architecture with avascular epithelium (EP) and vascularized deeper mucosa. LP, lamina propria. MM, muscularis mucosae. **i-k,** Another set of representative cross-sectional OCT (**i**), OPAM (**j**) and hybrid (**k**)

images of the healthy esophagus. **l-m,** Full-depth projection *en face* OCT (**l**) and OPAM (**m**) images. The OPAM image was generated utilizing the full bandwidth (3 – 110 MHz). **n,** Merged *en face* OPAM image combining the low and high frequency band images. Employing different colours to represent the low- and high-frequency components improved the identification of large and deeper vessels (low-frequency) and capillary loops (high-frequency).

In vivo imaging of swine esophagus involved placing the anesthetized animal on an elevation table (**Suppl Fig. 5a**), inserting a hollow mouth guard to prevent the mouth from closing (**Suppl Fig. 5b**), inserting the capsule through the mouth guard (**Suppl Fig. 5c-d**) into the distal esophagus, and pulling back the capsule to realize high-speed circumferential imaging of the esophagus (**Suppl Fig. 5e**) at 30 Hz frame rate. In the insertion process, the capsule was inserted by an operator manually. When the operator sensed a diminished tension from the capsule, it was estimated that the capsule had reached the stomach. The capsule was then withdrawn into the esophagus and the insertion process stopped. The real-time O2E cross-sectional visualization was also used to guide the insertion of the capsule.

Representative cross-sectional images (**Suppl Fig. 5f-k**) show that OCT image resolves the layered mucosal architecture, similar to results shown in the *ex vivo* imaging (**Suppl Fig. 4**). Note that the OCT imaging quality can be further improved by using a higher power on the sample. Currently the OCT setup uses two fiber couplers (**Suppl Fig. 1a**) to construct a dual-balanced Mach-Zehnder interferometer for generating OCT interference fringes. The first 50/50 fiber coupler divides ~65 mW optical power from the laser into 35.75 mW (measured value) for the sample arm and 30 mW (measured value) for the reference arm. With a 10/90 coupler in the sample arm, the incident power on the sample is ~5 mW, which is way under the safety limit (**Suppl Note 14**). By switching the 10/90 coupler to a 50/50 coupler, the incident power can be increased to ~17 mW and the signal-to-noise ratio can theoretically be enhanced by 2–3 dB. However, we kept using the low ~5 mW to avoid any potential photodamages to the *ex vivo* human esophageal resection samples in the clinical imaging, as the safety limit (**Suppl Note 14**) was tested only for skin and could mainly serve as a reference for the esophageal mucosa. The same power was then used for all experiments in this study. Replacing the fiber coupler with fiber optic circulators is an alternative way to deploy more power in the sample arm with the advantage of a high back-coupling efficiency. However, 1060 nm optical circulators are not as efficient as their 1300 nm counterparts. The typical insertion loss of low-loss 1060 nm fiber optic circulators is already 1.4–1.6 dB, equivalent to a loss of 30%. The circulators are also associated with polarization-induced ghost images and dispersion mismatches. In general, the quality of OCT imaging in the O2E setup can be improved by optimizing the imaging system, including increasing the incident power of OCT light at the tissue and using refined fiber optic components in the future.

On the other hand, OPAM reveals the mucosal vasculatures not visible in OCT structural imaging. Full-depth projection *en face* images (**Suppl Fig. 5l-m**) show a smooth mucosa (**Suppl Fig. 5l**) and delicate mucosal vasculatures (**Suppl Fig. 5m**). Compared with the full-bandwidth image (**Suppl Fig. 5m**), merging OPAM images reconstructed at low (3 – 40 MHz) and high frequency (40 – 110 MHz) bands (**Suppl Fig. 5n**) can better visualize different vascular features by separating the low-frequency LP vasculatures from the high-frequency capillary loops and small vessels.

6. Patient characteristics

A total of 14 patients suspected of BE neoplasia agreed to be recruited at Cambridge University Hospital for this study. After endoscopic inspections, a total of 10 patients received endoscopic mucosal resections. The characteristics of these 10 patients are listed in **Suppl Table. 1** below.

Supplementary Table.1 Characteristics of patients undertaken EMR

Number of patients	10
Average age	75 (70-88)
Sex (female/male)	3/7
Average BMI	25.8 (23-28.4)
Average Barrett's length	3.1 cm

7. EMR specimen imaging using O2E

Resected specimens were immediately pinned on silicon supports with 20-mm Agani needles. The silicon support was then mounted on a custom-designed specimen holder (**Suppl Fig. 6**) to maintain a uniform concave deformation of the support and the clinical specimen. The O2E capsule, which was mounted on a motorized stage (MTS50-Z8, Thorlabs, USA), was placed to gently contact the specimen and helically scan the specimen. Imaging finished within 40 s for a specimen.

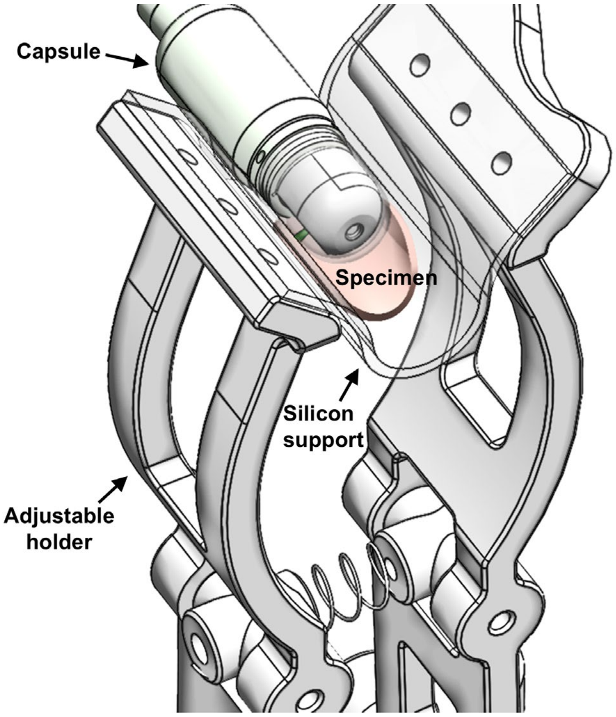

Supplementary Fig. 6| EMR specimen imaging holder. The silicon support was mounted on an adjustable holder to maintain the specimen in a concave form for imaging.

8. Correlating OPAM with CD31 immunostaining

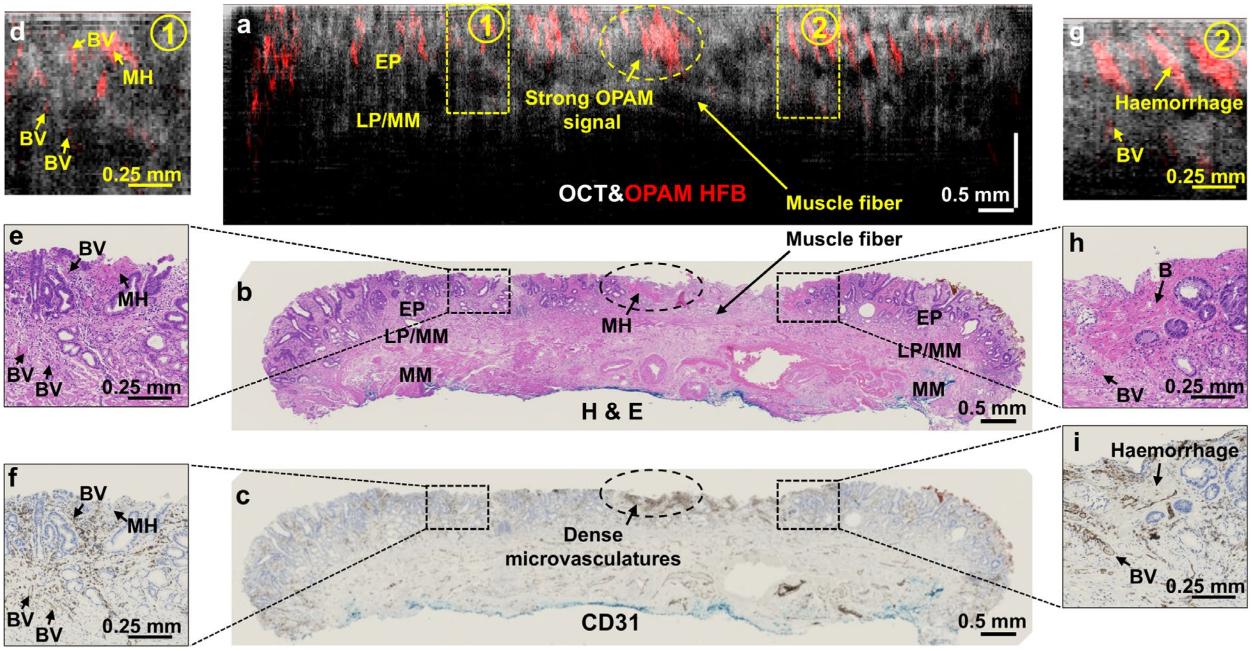

Supplementary Fig. 7| Correlating OPAM signals with CD31 immunostaining – case 1. a, A representative O2E cross-sectional image of an EMR specimen. EP, epithelium. LP, lamina propria. MM, muscularis mucosae. HFB, high-frequency band. **b & c**, Corresponding H&E and CD31 immunostaining images, respectively. **d-f**, Magnified O2E, H&E and CD31 immunostaining images corresponding to the encircled region #1 in **a**, respectively. BV, blood vessel. MH, micro-haemorrhage. **g - i**, Magnified O2E, H&E and CD31 immunostaining images corresponding to the encircled region #2 in **a**, respectively. B, blood.

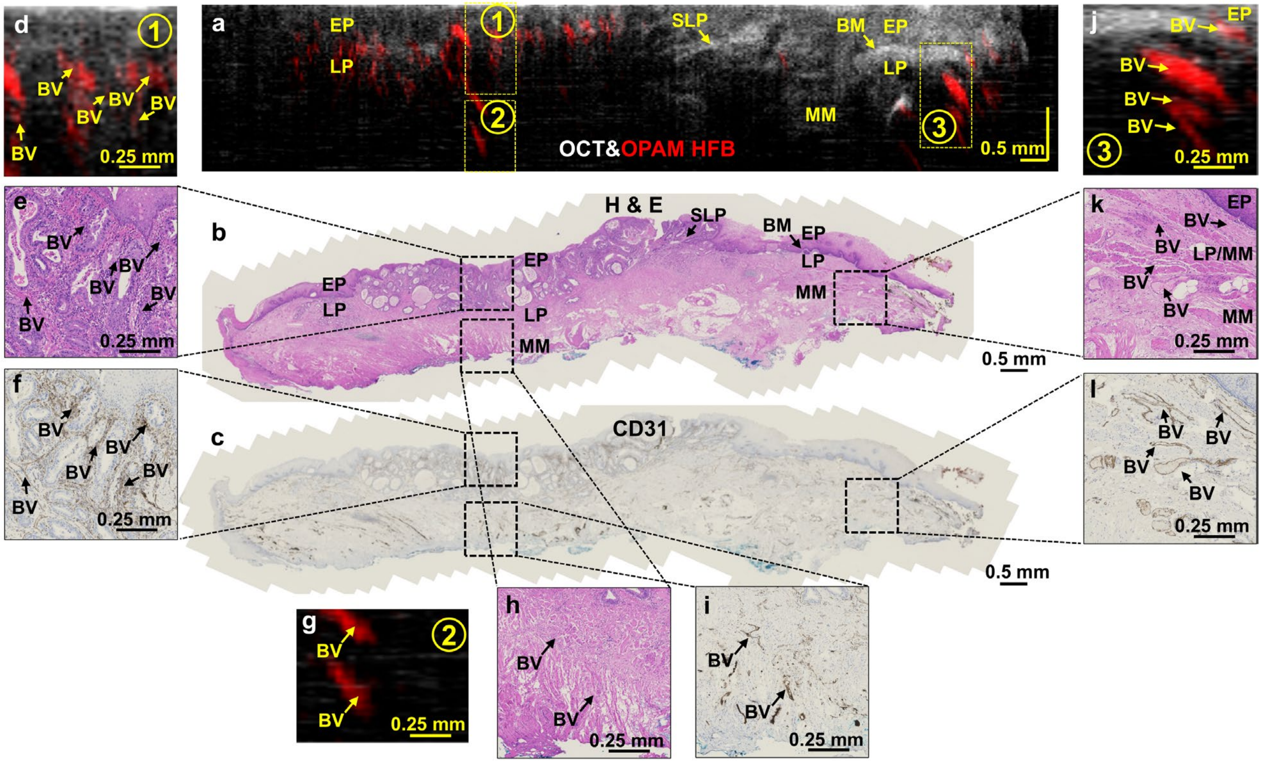

Supplementary Fig. 8| Correlating OPAM signals with CD31 immunostaining – case 2. a, Another representative O2E cross-sectional image of an EMR specimen. EP, epithelium. LP, lamina propria. MM, muscularis mucosae. BM, basement membrane. SLP, superficial lamina propria. HFB, high-frequency band. **b & c**, Corresponding H&E and CD31 immunostaining images, respectively. **d-f**, Magnified O2E, H&E and CD31 immunostaining images corresponding to the encircled region #1 in **a**, respectively. BV, blood vessel. **g-i**, Magnified O2E, H&E and CD31 immunostaining images corresponding to the encircled region #2 in **a**, respectively. **j-l**, Magnified O2E, H&E and CD31 immunostaining images corresponding to the encircled region #3 in **a**, respectively.

Correlating O2E cross-sectional images with H&E slides has shown that OPAM signals correlate well with the visible blood in histology (e.g. **Fig. 2**). Imaging of *ex vivo* swine esophagus with reduced blood content shows minimal OPAM signals from other chromophores inside the mucosa (**Suppl Fig. 4**), thus further proving that blood is the main absorber in the esophageal mucosa at 532 nm wavelength. However, it is not known whether OPAM images of *ex vivo* EMR samples represent the actual vascular morphology, as blood vessels are often not visible in H&E slides and haemorrhages can be induced in the resection. Therefore, a total of 17 sections from EMR specimens were stained with a CD31 immunostain specific to vascular epithelial cells to reveal the vasculatures within the mucosa. CD31 results were then compared with O2E cross-sectional images and H&E images. **Suppl Fig. 7** & **8** show representative co-registered O2E, H&E and CD31 images. By rendering the vascular epithelial cells in brown, CD31 immunostaining clearly shows the vascular distributions inside the mucosa. OPAM signals matches the location of these blood vessels well, e.g. the strong OPAM signal in **Suppl Fig. 7a** accurately reflects the dense vasculatures found in the CD31 image (**Suppl Fig. 7c**). Magnified OPAM, H&E and CD31 images (**Suppl Fig. 7d-i, Suppl Fig. 8d-l**) further demonstrate high-quality correlations between OPAM signals and mucosal vasculatures. The results prove that OPAM signals are mainly generated from blood within mucosal vasculatures. However, comparison of H&E and CD31 images also clearly shows the existence of haemorrhages in the specimens, possibly induced by the resection process. For example, in **Suppl Fig. 7e**, a patch of blood can be clearly observed by H&E staining. But through CD31 immunostaining (**Suppl Fig. 7f**), it is proven to be a location of micro-haemorrhages as no blood vessels are found in this region. In **Suppl Fig. 7d**, it is shown that the aforementioned micro-haemorrhages produce prominent OPAM signals. Significant OPAM signals produced from haemorrhages can also be seen in **Suppl Fig. 7g**. Therefore, the vascular morphology revealed by OPAM in *ex vivo* EMR studies was masked by the unavoidable haemorrhage induced in the resection process. Correlating OPAM with H&E and CD31 (**Suppl Fig. 8g-i**) also confirms that OPAM is able to image deep vasculatures of ~1.5 mm depth inside the muscularis mucosae layer.

9. Analysis of vessel diameter and orientation through cross-sectional images

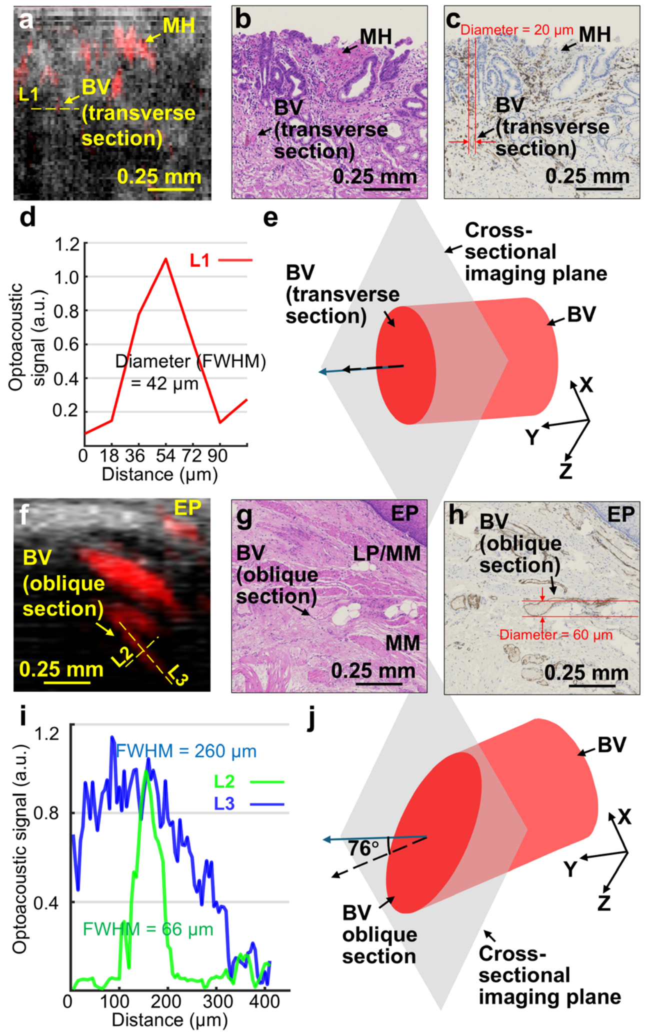

Supplementary Fig. 9| Cross-sectional images enable analysis of blood vessel metrics, including vessel diameter and vessel orientation. a-c, Representative O2E cross-sectional, H&E and CD31 images of a small portion of dysplastic mucosa. CD31 shows the transverse section of a small vessel with a diameter of 20 µm. The same vessel can be seen in the O2E cross-sectional image. BV, blood vessel. MH, micro-haemorrhage. **d**, The OPAM line-profile (L1 in **a**) of the annotated blood vessel in **a-c**. FWHM, full-width-half-maximum. **e**, Illustration of the annotated blood vessel in **a-c** orienting perpendicularly to O2E cross-sectional imaging plane. The long blue arrow denotes the directional vector of the blood vessel. The long black arrow denotes the normal vector of the cross-sectional imaging plane. **f-h**, Representative O2E cross-sectional, H&E and CD31 images of a small portion of normal squamous mucosa. CD31 shows the oblique section of a vessel with a diameter of 60 µm. The oblique section of the same vessel can be seen in the O2E cross-sectional image as an elongated ellipse. EP, epithelium. LP, lamina propria. MM, muscularis mucosae. **i**, OPAM line-profiles corresponding to the major (L3 in **f**) and minor (L2 in **f**) axis of the elliptic oblique section of the annotated blood vessel in **f-h**. **j**, Illustration of the annotated blood vessel in f-**h** orienting non-perpendicularly to O2E cross-sectional imaging plane. As the lengths of the major and minor axes are 260 and 66 µm, respectively, the angle between the directional vector of the blood vessel and the normal vector of the cross-sectional imaging plane can be determined as 76°. The long blue arrow denotes

the directional vector of the blood vessel. The long black arrow denotes the normal vector of the cross-sectional imaging plane.

Correlating O2E cross-sectional images with CD31 results not only show that OPAM signal originates from mucosal vasculatures, but also demonstrate that OPAM cross-sectional images accurately reconstruct blood vessel cross-sections. For blood vessels oriented perpendicularly to the cross-sectional imaging plane (**Suppl Fig. 9a-c**), the transverse sections of the vessels are shown as a round dot in cross-sectional images. For a small vessel of 20 µm diameter, due to the insufficient lateral resolution of OPAM, the resolved diameter is 42 µm according to the full-width-half-maximum distance of the vessel cross-section (**Suppl Fig. 9d**). **Suppl Fig. 9e** then illustrates the orientation of this vessel to the cross-sectional imaging plane. For blood vessels oriented non-perpendicularly to the cross-sectional imaging plane (**Suppl Fig. 9f-h**), the oblique sections of the vessels are displayed as elongated elliptic areas in cross-sectional images (**Suppl Fig. 9f**). OPAM allows measurement of major and minor axis length of the elliptic oblique vessel section (**Suppl Fig. 9i**). The lengths of the major and minor axes are determined as 260 and 66 µm, respectively, according to the OPAM line profiles (**Suppl Fig. 9i**). The measured vessel diameter is then 66 µm, close to the value of 60 µm determined in the CD31 image (**Suppl Fig. 9h**). The orientation of the blood vessel in accordance with the cross-sectional imaging plane can then be determined as the angle between the directional vector of the blood vessel and the normal vector of the cross-sectional imaging plane (**Suppl Fig. 9j**). In this case, the angle is calculated as 76° by deriving the arctan value of the ratio of major axis length to the minor axis length.

10. High-frequency band OPAM images of EMR specimens

We show in this section that the use of high-frequency band OPAM images are critical in resolving detailed mucosal vascular morphology. Representative vascular patterns in gastric metaplastic, intestinal metaplastic and dysplastic mucosae resolved by low-frequency band (LFB, 3 – 40 MHz), full-frequency band (FFB, 3 – 110 MHz) and high-frequency band (HFB, 40 – 110 MHz) OPAM are illustrated in *en face* OPAM images (**Suppl Fig. 10 a-d**). OPAM images reconstructed at FFB and LFB appear similar (**Suppl Fig. 10 a-d**). Line profiles in **Suppl Fig. 10e** shows that this is because the low-frequency components of the optoacoustic signals are much larger in signal amplitude than the high-frequency components. Conversely, HFB OPAM images (**Suppl Fig. 10 a-d**) show fine details of vasculatures with improved resolutions than FFB and LFB OPAM, due to the elimination of strong low-frequency signals.

Normalized OPAM line profiles in **Suppl Fig. 10f** demonstrate a good matching between vascular and mucosal patterns. Areas with strong OCT signals (**Suppl Fig. 10f**) are superficial connective tissues. Areas with low OCT signals (**Suppl Fig. 10f**) correspond to crypts, glands or pits. **Suppl Fig. 10f** shows that most of the time, OPAM LFB, FFB, HFB correctly visualize the vascularization in the connective tissues and the avascular nature of cryptic and glandular structures. However, as can be seen in L5 of **Suppl Fig. 10f**, only the HFB line profile precisely shows the vascularization around the pits. An improved resolution of OPAM HFB can be seen in these line profiles (**Suppl Fig. 10f**).

To quantify the actual resolution improvement with OPAM HFB, the OPAM data of the edge of a resolution target in water (**Suppl Note 1**) was reconstructed at LFB, FFB and HFB, respectively. The same steps as **Suppl Note 1** were followed to derive line spread functions (LSF) at these 3 frequency bands. From the corresponding FWHMs of the LSFs, the lateral resolutions of OPAM LFB, FFB and HFB were estimated as 56 µm, 30 µm and 21 µm, respectively (**Suppl Fig. 10g**). The axial resolutions of OPAM LFB, HFB and HFB were obtained by frequency filtering A-scan data of the 7-µm diameter carbon fiber at the corresponding frequency bands. After Hilbert transform of these A-scans, the axial resolutions of OPAM LFB, FFB and HFB were estimated as 38 µm, 17 µm and 12 µm, respectively (**Suppl Fig. 10h**).

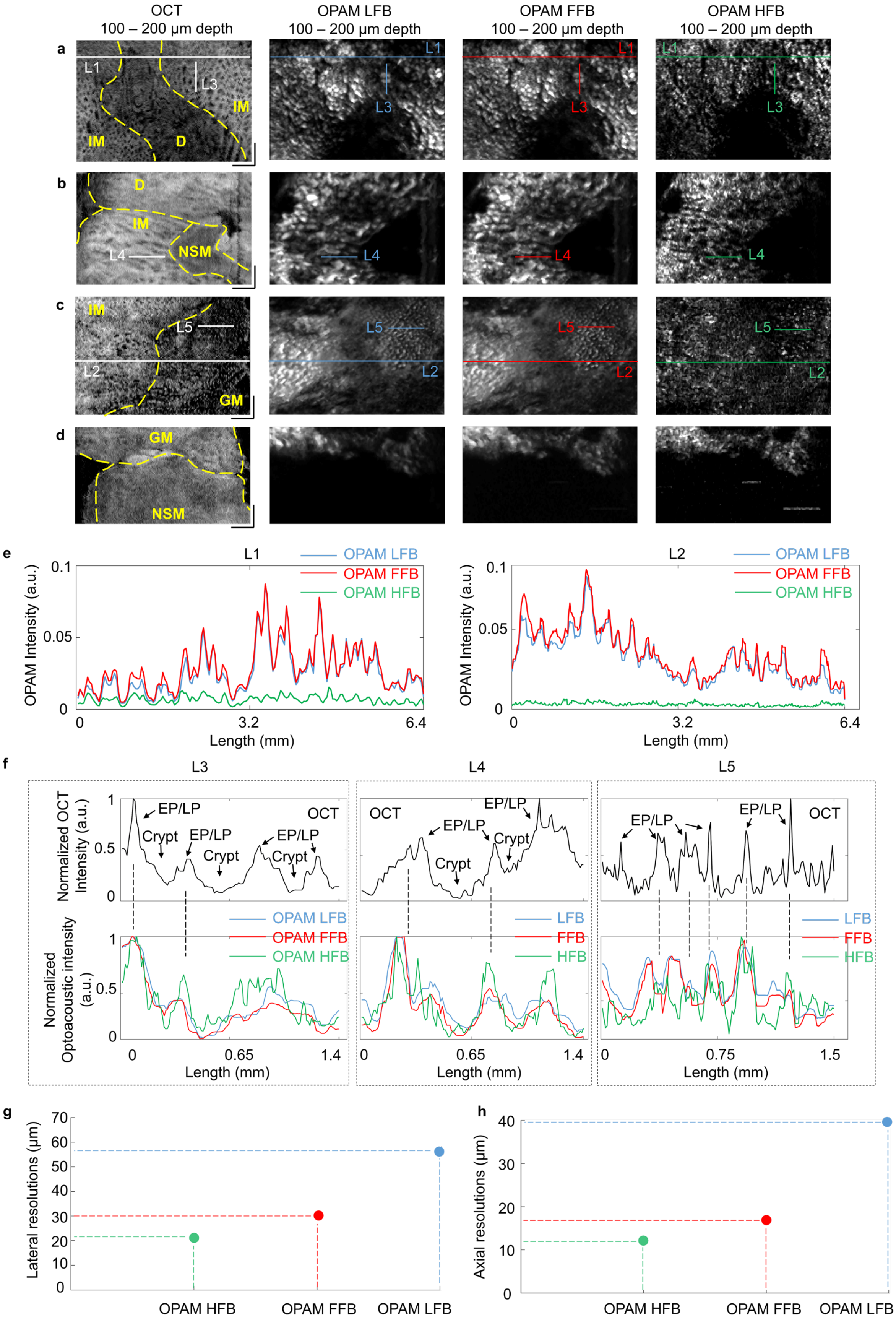

OCT
100 – 200 µm depth
OPAM LFB
100 – 200 µm depth
OPAM FFB
100 – 200 µm depth
OPAM HFB
100 – 200 µm depth
a
L1
L3
IM
IM
D
b
D
IM
NSM
L4
c
IM
L5
L2
GM
d
GM
NSM
e
L1
OPAM LFB
OPAM FFB
OPAM HFB
OPAM Intensity (a.u.)
0.1
0.05
0
3.2
6.4
Length (mm)
L2
f
L3
L4
L5
Normalized OCT Intensity (a.u.)
EP/LP
Crypt
OCT
Normalized Optoacoustic intensity (a.u.)
LFB
FFB
HFB
0.65
1.4
0.75
1.5
g
Lateral resolutions (µm)
70
60
50
40
30
20
10
OPAM HFB
OPAM FFB
OPAM LFB
h
Axial resolutions (µm)

Supplementary Fig. 10| Frequency-banding OPAM images of EMR specimens. a-d, Representative images showing the epithelium (EP) and vascular pattern in NSM, GM, IM and dysplastic regions using OCT 100 – 200 µm depth projection *en face* images and OPAM 100 – 200 µm depth projection *en face* images at LFB, FFB and HFB. Scale bars: 1 mm. NSM, normal squamous mucosa. GM, gastric metaplasia. IM, intestinal metaplasia. D, dysplasia. LFB, low-frequency band. FFB, full-frequency band. HFB, high-frequency band. **e**, Line profiles show that LFB components are the major components of OPAM signals. **f**, Line profiles show that the vascular pattern resolved by OPAM matches the mucosal pattern in OCT. However, in L5 it is clear only HFB OPAM precisely images the vasculatures around the gastric pits. It can also be seen that HFB offers improved resolutions. LP, lamina propria. **g & h**, Lateral and axial resolutions of HFB OPAM, FFB OPAM and LFB OPAM characterized by filtering the optoacoustic signals at the resolution target edge in **Suppl Note 1** at HFB, FFB and LFB, respectively.

11. Quantification of mucosal inhomogeneity

Suppl Fig. 11a illustrates the steps taken to calculate the mucosal inhomogeneity within a selected ROI. **Suppl Fig. 11 b & c** uses an intestinal metaplasia region to illustrate these steps. With the OCT 100 – 200 µm depth projection *en face* image, the boundaries of an ROI were manually defined in MATLAB using function roipoly (**Suppl Fig. 11b**), in accordance with histopathology. The next step was to separate the ROI into 2×3 mm^2 rectangular grids (**Suppl Fig. 11c**) and calculate the local coefficient of variation within each grid according to

$$C_v = \frac{\sigma}{\mu},$$

where μ and σ are the mean pixel value and standard deviation of pixel values, respectively. Only complete grid cells were included for quantification. The mean value of the obtained local coefficient of variations was defined as the mucosal inhomogeneity of the ROI.

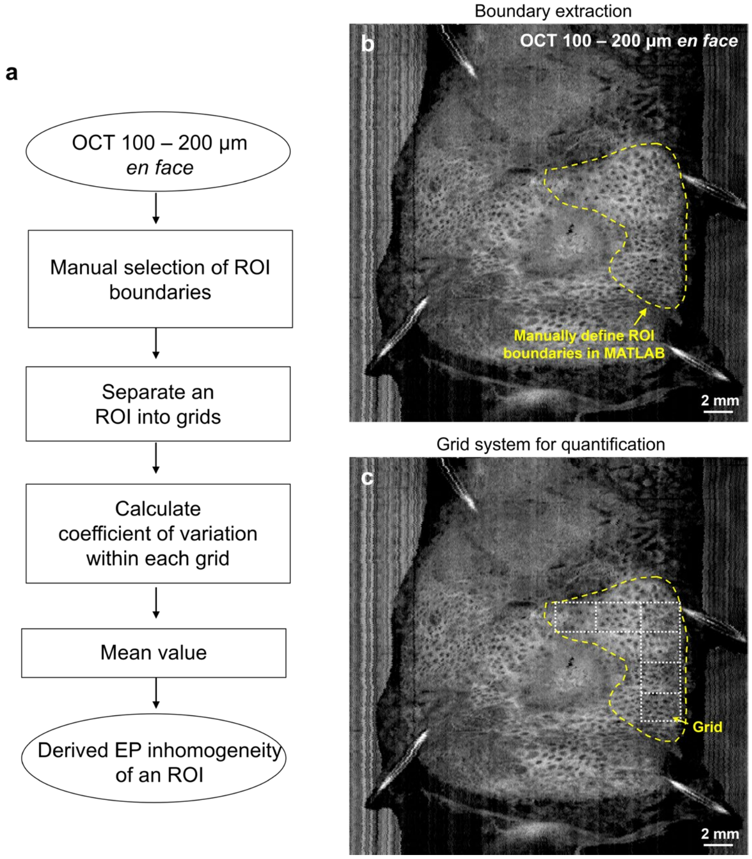

Supplementary Fig. 11| Quantification of mucosal inhomogeneity. **a**, A flowchart shows the steps for quantifying mucosal inhomogeneity in each ROI. **b**, Illustration of manual extraction of the boundary of an intestinal metaplasia region according to the histopathology. Dashed yellow lines represent the boundary of this ROI. **c**, Illustration of using 2×3 mm^2 grids to divide an ROI into multiple grid cells and calculating coefficient of variation within each grid cell independently. Dashed white lines illustrate the grids for the calculation.

12. Development of O2E classification system and its validation

A flowchart-based O2E classification system was developed to aid graders in the classification tests based on cross-sectional and *en face* O2E features. The classification system includes 2 flowchart algorithms for the 2 corresponding classification tests. The first flowchart (**Suppl Fig. 12**) was designed to aid graders in classification Test 1 to classify each ROI as either abnormal (metaplastic, dysplastic or cancerous mucosa) or normal (normal squamous mucosa). In OCT step of Test 1, graders rated each ROI by observing whether the epithelium was homogeneous using OCT 100 – 200 µm *en face* images. In Hybrid step of Test 1, graders reconsidered their ratings by observing OCT 100 – 200 µm and OPAM *en face* images side by side. If the graders find the epithelium to be inhomogeneous or the area contains abnormally shallow vasculatures (through depth-coding, these abnormally shallow vasculatures are rendered in green and yellow colors), graders should classify the ROI as an abnormal area according to the flowchart.

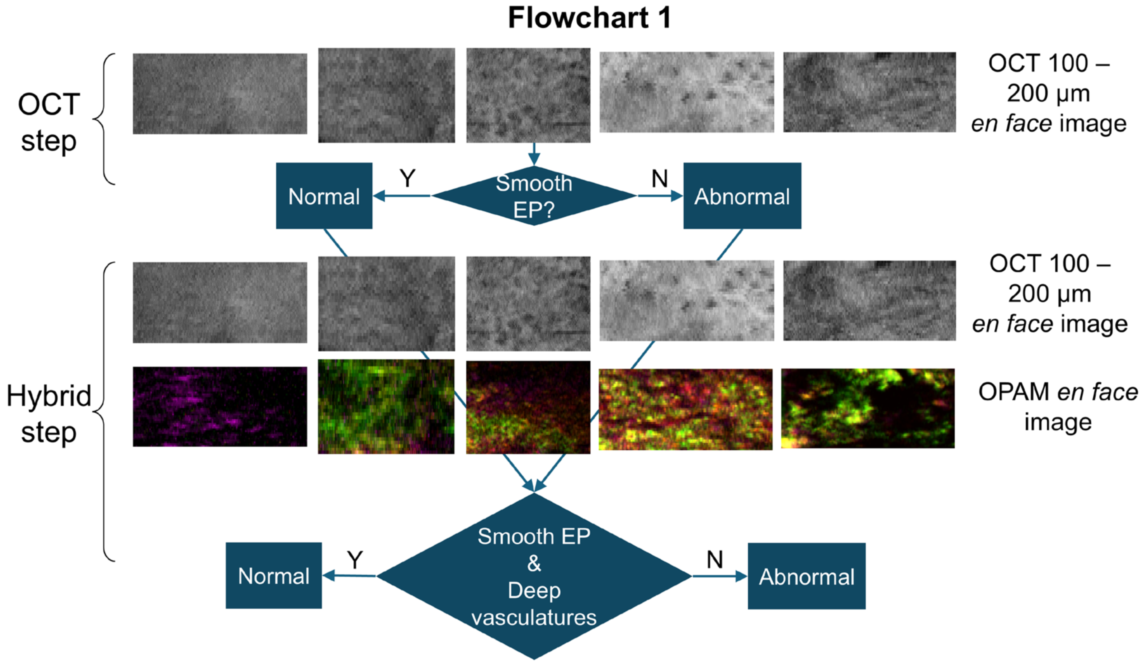

Supplementary Fig. 12| O2E classification system – flowchart 1. Flowchart 1 illustrates stepwise classification of ROIs in Test 1 as normal or abnormal areas based on *en face* O2E features. In OCT step of Test 1, graders followed the flowchart to observe whether the epithelium of the ROI was homogeneous based on OCT 100 – 200 µm *en face* images. In Hybrid step, graders followed the flowchart to observe whether the epithelium was homogeneous or if the mucosa contained abnormally shallow vasculatures using OCT 100 – 200 µm and OPAM *en face* images side by side. EP, epithelium.

The second flowchart (**Suppl Fig. 13**) was designed to guide graders in classification Test 2 to determine the exact mucosal type of ROIs in a stepwise manner. In OCT step of Test 2, graders made ratings with OCT 100 – 200 µm *en face* and OCT cross-sectional images side by side. Depending on the size of the ROI, a number of 1 to 4 OCT cross-sectional images per ROI were provided for the graders. In Hybrid step of Test 2, OPAM *en face* images are additionally provided to graders. Graders used OCT 100 – 200 µm *en face* image, OCT cross-sectional images and OPAM *en face* images simultaneously to reconsider their ratings made in the previous OCT step. Graders were advised to strictly follow the flowchart to make their ratings.

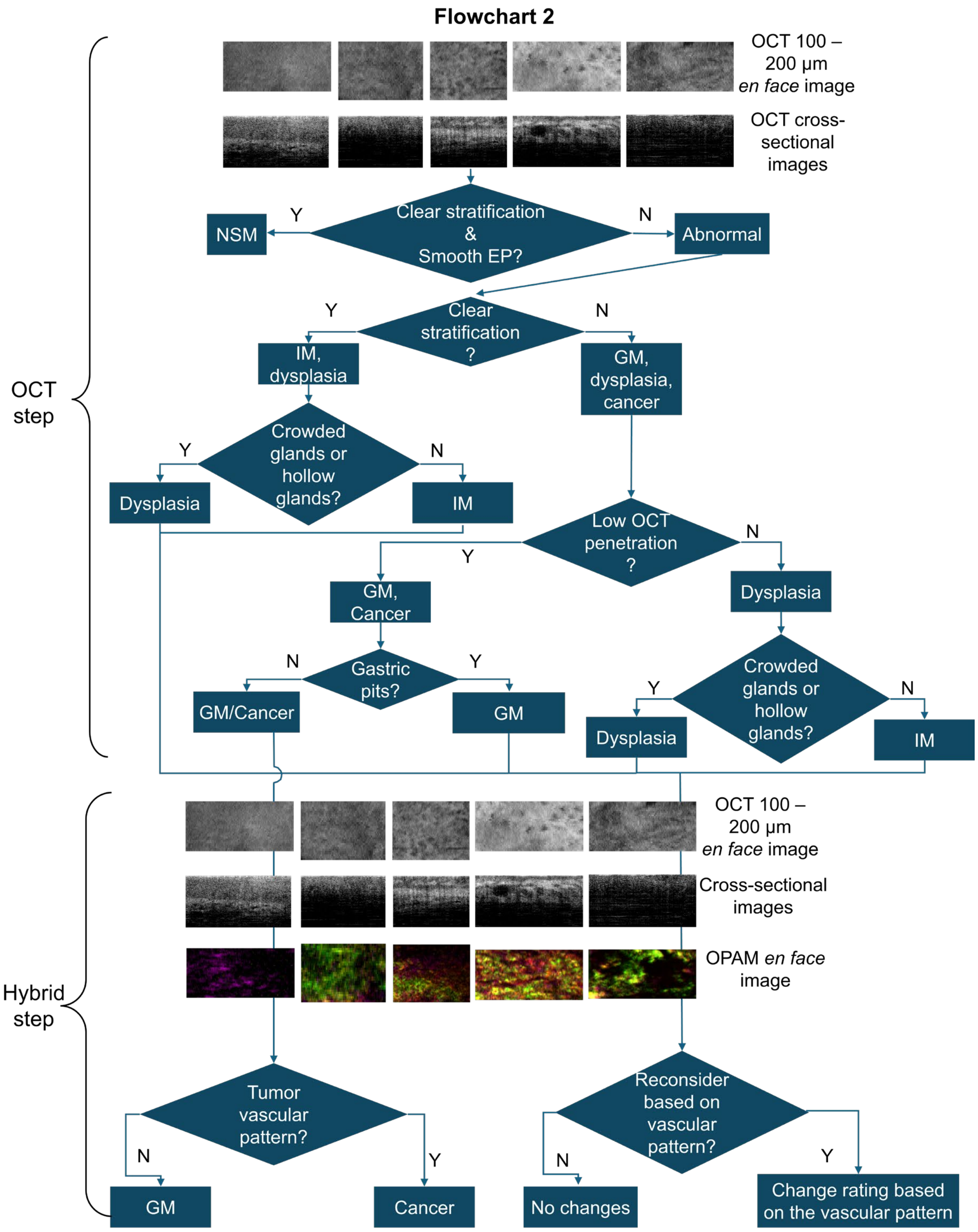

Supplementary Fig. 13| O2E classification system – flowchart 2. Flowchart 2 provided stepwise guides to aid graders in using the *en face* and cross-sectional O2E features to distinguish ROIs into normal squamous mucosa (NSM), gastric metaplasia (GM), intestinal metaplasia (IM), dysplasia or intra-mucosal cancer. Flowchart 2 was used in classification Test 2. EP, epithelium.

13. Quantification of O2E features in gastric metaplasia (GM) type 1, GM type 2, low-grade dysplasia (LGD) and high-grade dysplasia (HGD)

In the *ex vivo* O2E imaging study of BE specimens with histopathological validations, it was found that based on whether prominent pit compartments were imaged in OCT, gastric metaplastic ROIs could be separated into gastric metaplasia (GM) type 1 and GM type 2. The GM type 1 shows obvious gastric pit structures in OCT imaging. GM type 2 shows a homogeneous subsurface architecture similar to intramucosal cancer (IMC) in OCT. In the study, low-grade dysplasia (LGD) and high-grade dysplasia (HGD) were categorized as a dysplasia group despite that the pathologist graded the dysplastic cases as LGD or HGD in the histopathological analysis. In this section, the cross-sectional and *en face* O2E features of GM type 1, GM type 2, LGD and HGD ROIs are calculated and compared with other mucosal types.

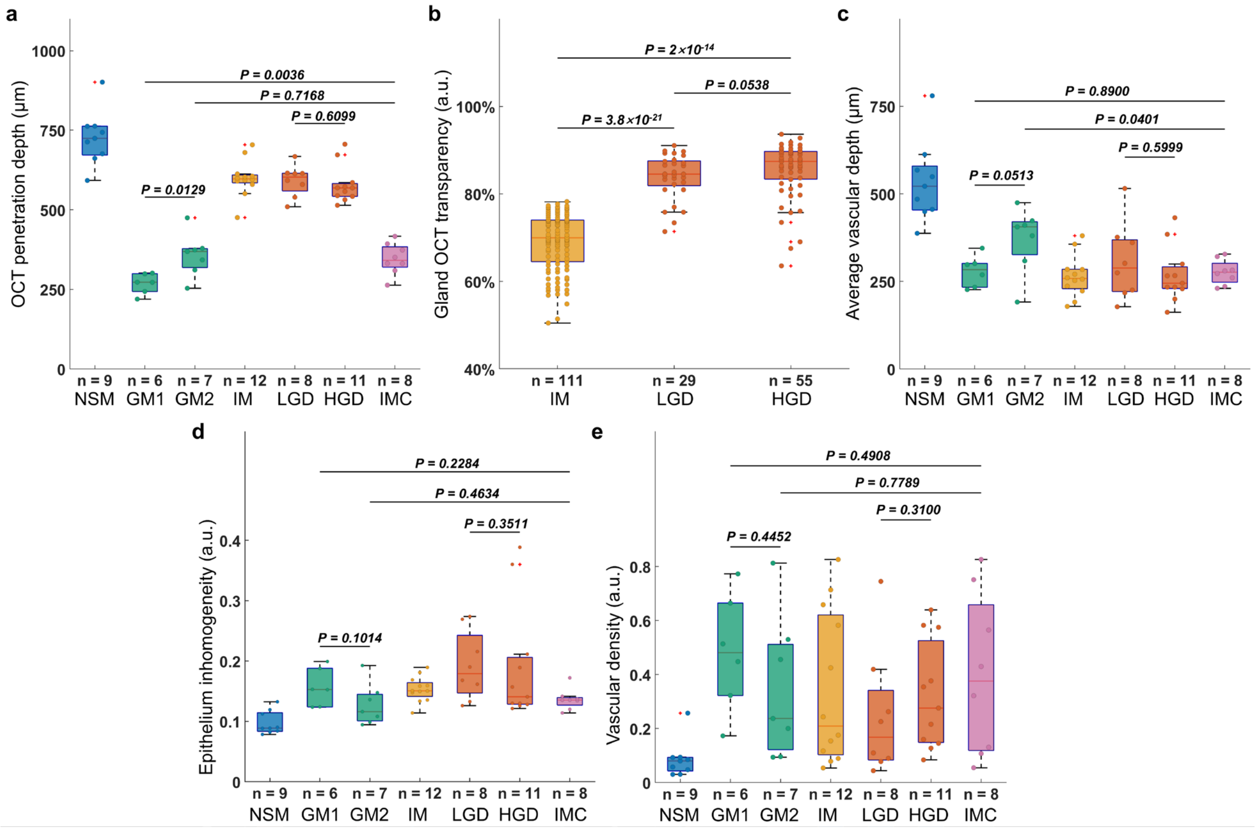

Supplementary Fig. 14| Quantification of cross-sectional and *en face* O2E features in GM type 1, GM type 2, LGD and HGD ROIs. a, Calculated OCT penetration depths in normal squamous mucosa (NSM), GM type 1, GM type 2, intestinal metaplasia (IM), LGD, HGD and intra-mucosal cancer (IMC) ROIs. **b**, Calculated OCT transparency of epithelial glands extracted from IM, LGD and HGD ROIs, respectively. **c**, Calculated average depths of blood vessels imaged through OPAM in NSM, GM type 1, GM type 2, IM, LGD, HGD and IMC ROIs. **d**, Calculated epithelial inhomogeneity in NSM, GM type 1, GM type 2, IM, LGD, HGD and IMC ROIs. **e**, Calculated density of blood vessels imaged through OPAM in NSM, GM type 1, GM type 2, IM, LGD, HGD and IMC ROIs. The differences between samples were estimated using two-sided Wilcoxon rank sum tests. On each box in the box and whisker plots, the central mark indicates the median, and the bottom and top edges of the box indicate the 25th and 75th percentiles, respectively. The whiskers extend to the most extreme data points not considered outliers, and the outliers are plotted individually using the '+' marker symbol.

The results (**Suppl Fig. 14a**) show that OCT penetration is significantly lower in GM type 1 than GM type 2 (*P*=0.0129, two-sided Wilcoxon rank sum tests, same below) and IMC (*P*=0.0036). OCT penetration is not significantly different between GM type 2 and IMC (*P*=0.7168), corroborating the finding that GM type 2 and IMC are difficult to distinguish using OCT. The OCT penetration is also not significantly different between LGD and HGD (*P*=0.7168). The calculated OCT transparency of glands extracted from IM, LGD and HGD ROIs (**Suppl Fig. 14b**) shows that while epithelial glands in LGD and HGD are significantly more transparent to OCT than glands in IM (*P*<0.001), epithelial glands in LGD and HGD are not significantly different in their OCT transparency (*P*=0.0538). Quantification of the depths of the imaged vasculatures in OPAM (**Suppl Fig. 14c**) shows no significant difference between LGD and HGD (*P*=0.5999). However, while vascular depths in GM type 1 are not significantly different from that in IMC (*P*=0.89), the imaged vasculatures in GM type 2 are significantly deeper than that in IMC (*P*=0.04), suggesting again the importance of OPAM features for distinguishing between GM type 2 and IMC. Quantification of epithelial inhomogeneity (**Suppl Fig. 14d**) shows no significant difference between either GM type 1 and GM type 2 (*P*=0.1014), or between LGD and HGD (*P*=0.3511). Likewise, quantification of the density of imaged vasculatures in all ROIs (**Suppl Fig. 14e**) shows no significant difference between either GM type 1 and GM type 2 (*P*=0.4452), or between LGD and HGD (*P*=0.31). In general, quantification of cross-sectional and *en face* O2E features demonstrates no significant differences between LGD and HGD ROIs, as grading of LGD and HGD is often dependent on the degree of observed cytologic atypia[4], which are not resolvable in O2E due to the resolution limit. It should also be noted that as dysplasia grading is known to include substantial interobserver variability[4,5], the dysplasia grading presented in this study could be biased.

14. Laser safety limit

Due to a lack of scientific data on the laser safety level on esophageal tissues, we base our safety requirements on laser radiation of the human skin. The OCT and OPAM laser spot sizes on the outer surface of the capsule were measured as 50 and 500 µm, respectively. As the OCT and OPAM laser spots on the tissue surface are smaller than the 3.5 mm diameter limiting aperture defined in the *American National Standard for Safe Use of Lasers* (ANSI Z136.1-2014, Laser Institute of America), we used the defined limiting aperture for hazard evaluation. With a capsule diameter of 12.5 mm and a rotational speed of 30 Hz (188.5 rad/s), each time the capsule scans across the aperture, the exposure time for OCT and OPAM on the limiting aperture was:

$$\mathrm{t} = \frac{Limiting\ aperture\ (rad)}{Rotational\ speed\ (\frac{rad}{s})} = \frac{0.56}{188.5} = 3\ ms$$

Taking the laser spot sizes into consideration, the applied fluences of OCT and OPAM were:

$$E_{OCT} = \frac{5\ (\mathrm{mW})}{\pi \times 0.35 \times 0.005\ (cm^2)} \times \mathrm{t} = 2.7 \frac{mJ}{cm^2},$$

$$E_{OAT} = \frac{0.012\ (\mathrm{mJ})}{\pi \times 0.35 \times 0.05\ (cm^2)} = 0.22 \frac{mJ}{cm^2},$$

respectively.

At a central wavelength of 1060 nm, the maximum permissible exposure (MPE) for OCT would be

$$MPE_{OCT} = 1.1 \times t^{0.25} = 257 \frac{mJ}{cm^2}.$$

Therefore, the OCT exposure was well below the MPE.

For OPAM with repetitively pulsed lasers, the MPE would be the smallest MPE value of MPEs determined by Rules 1 and 2:

$$MPE = \min[MPE_{rule1}, MPE_{rule2}].$$

With a pulse duration of 2 ns and an operating wavelength of 532nm,

$$MPE_{rule1} = 20 \frac{mJ}{cm^2}.$$

MPE Rule 2 (Average Power rule): The average MPE per pulse distributed over a number of laser pulses over the exposure time would be:

$$MPE_{rule2} = \frac{MPE_T}{N},$$

where N is the number of pulses during the exposure period given by the product of the pulse repetition rate (PRR) and exposure time. With a wavelength of 532 nm, the maximum radiant exposure MPE_T, for an exposure time between 100 ns to 10 s would be

$$MPE_T = 1.1 \times t^{0.25} = 257 \frac{mJ}{cm^2}.$$

Therefore, the average MPE per pulse distribution as a function of PRR and exposure time was given as follows:

$$MPE_{rule2} = \frac{257}{Int(PRR \times \mathrm{t})} \frac{mJ}{cm^2}.$$

With a PRR of 42.5kHz, therefore

$$MPE_{rule2} = 1.93 \frac{mJ}{cm^2}.$$

Hence, the MPE restricting our application was 1.93 mJ/cm^2. With a fluence of 0.22 mJ/cm^2, OPAM did not exceed the safety limit.

References

1. Aguirre, J. *et al.* Precision assessment of label-free psoriasis biomarkers with ultra-broadband optoacoustic mesoscopy. *Nat Biomed Eng* **1**, 68 (2017).

2. Cubeddu, R., Pifferi, A., Taroni, P., Torricelli, A. & Valentini, G. A solid tissue phantom for photon migration studies. *Phys Med Biol* **42**, 1971–1979 (1997).

3. Sweer, J. A., Chen, M. T., Salimian, K. J., Battafarano, R. J. & Durr, N. J. Wide-field optical property mapping and structured light imaging of the esophagus with spatial frequency domain imaging. *J Biophotonics* **12**, (2019).

4. Bujanda, D. E. & Hachem, C. Barrett's Esophagus: A Comprehensive and Contemporary Review for Pathologists. *Am J Surg Pathol* **40**, 211–213 (2016).

5. Odze, R. D. Diagnosis and grading of dysplasia in Barrett's oesophagus. *J Clin Pathol* **59**, 1029–1038 (2006).
