# Supplementary material for: Tethered optoacoustic and optical coherence tomography capsule endoscopy for label-free assessment of Barrett’s oesophageal neoplasia
Source: Nat Biomed Eng. 2025 Aug 6;10(2):259–76. doi: 10.1038/s41551-025-01462-0 (PMC12920095; doi:10.1038/s41551-025-01462-0)
Supplement: Supplementary file 2 — Reporting Summary [file 41551_2025_1462_MOESM2_ESM.pdf]

nature portfolio

Corresponding author(s): Vasilis Ntziachristos

Last updated by author(s): Apr 29, 2025

Reporting Summary

Nature Portfolio wishes to improve the reproducibility of the work that we publish. This form provides structure for consistency and transparency in reporting. For further information on Nature Portfolio policies, see our Editorial Policies and the Editorial Policy Checklist.

Statistics

For all statistical analyses, confirm that the following items are present in the figure legend, table legend, main text, or Methods section.

n/a	Confirmed	
☐	☒	The exact sample size (*n*) for each experimental group/condition, given as a discrete number and unit of measurement
☐	☒	A statement on whether measurements were taken from distinct samples or whether the same sample was measured repeatedly
☐	☒	The statistical test(s) used AND whether they are one- or two-sided<br>*Only common tests should be described solely by name; describe more complex techniques in the Methods section.*
☒	☐	A description of all covariates tested
☒	☐	A description of any assumptions or corrections, such as tests of normality and adjustment for multiple comparisons
☐	☒	A full description of the statistical parameters including central tendency (e.g. means) or other basic estimates (e.g. regression coefficient) AND variation (e.g. standard deviation) or associated estimates of uncertainty (e.g. confidence intervals)
☐	☒	For null hypothesis testing, the test statistic (e.g. *F*, *t*, *r*) with confidence intervals, effect sizes, degrees of freedom and *P* value noted<br>*Give P values as exact values whenever suitable.*
☒	☐	For Bayesian analysis, information on the choice of priors and Markov chain Monte Carlo settings
☒	☐	For hierarchical and complex designs, identification of the appropriate level for tests and full reporting of outcomes
☒	☐	Estimates of effect sizes (e.g. Cohen's *d*, Pearson's *r*), indicating how they were calculated

Our web collection on statistics for biologists contains articles on many of the points above.

Software and code

Policy information about availability of computer code

Data collection	The O2E imaging datasets were collected using a licensed custom Microsoft Windows (Windows 10 64 bit) application.
Data analysis	Offline OCT reconstruction was implemented via custom-built LabVIEW code (LabVIEW 2016), which can be provided for research purposes from the corresponding authors on reasonable request. OPAM reconstruction and O2E data analysis were implemented via custom-built MATLAB code (MATLAB 2018a). The MATLAB code is available via Zenodo at https://doi.org/10.5281/zenodo.10817674.

For manuscripts utilizing custom algorithms or software that are central to the research but not yet described in published literature, software must be made available to editors and reviewers. We strongly encourage code deposition in a community repository (e.g. GitHub). See the Nature Portfolio guidelines for submitting code & software for further information.

Data

Policy information about availability of data

All manuscripts must include a data availability statement. This statement should provide the following information, where applicable:

- Accession codes, unique identifiers, or web links for publicly available datasets
- A description of any restrictions on data availability
- For clinical datasets or third party data, please ensure that the statement adheres to our policy

The datasets analysed during the current study are attached. O2E patient data is too large to be publicly shared, yet it is available for research purposes from the corresponding authors on reasonable request. O2E data of healthy human labial mucosa is available via Zenodo at https://doi.org/10.5281/zenodo.10817674.

Research involving human participants, their data, or biological material

Policy information about studies with human participants or human data. See also policy information about sex, gender (identity/presentation), and sexual orientation and race, ethnicity and racism.

Reporting on sex and gender	This study included human esophageal tissues yielded by endoscopic submucosal resections from 7 male and 3 female patients suspected of esophageal dysplasia or intrmucosal cancer. The study was designed to research O2E imaging features of esophageal dysplasia and cancer. Although esophageal cancer is a male predominant disease, there is no evidence that sex impacts the morphological and prognostic features of this disease, hence the male predominance in our human data is not expected to introduce any bias in the analysis of these human tissues.
Reporting on race, ethnicity, or other socially relevant groupings	Race, ethnicity or other socially relevant groupings were not considered with regards to the analysis and conclusions of this study, as there is no evidence that these factors impacts the morphological features of Barrett's esophagus, dysplasia or intramucosal esophageal cancer.
Population characteristics	The age of the patients ranged from 70 to 88 years old at the time of tissue collection. All patients had confirmed Barrett's esophagus and were enrolled in the endoscopic surveillance program. In endoscopic inspections, all patients were suspected of Barrett's neoplasia.
Recruitment	We recruited 14 patients with suspected Barrett's neoplasia in endoscopic inspections at Cambridge University Hospital. From these patients, esophageal tissues containing suspicious lesions were obtained and used for this study from 7 male and 3 female patients, due to inappropriateness of endoscopic mucosal resections on other patients. As all patients possessed visually suspicious lesions, the recruitment may overestimate the ability of O2E to visualize Barrett's neoplasia, as some lesions can be visually inconspicuous. However, as demonstrated in this study, O2E reveals heterogeneous mucosal types irregularly embedded in the resected esophageal tissue, covering all stages in the progression from normal mucosa to Barrett's neoplasia.
Ethics oversight	Written informed consent was obtained from each patient under an approved ethics protocol (18/EM/0069) by East Midlands - Nottingham 2, Research Ethics Committee of The National Health Service.

Note that full information on the approval of the study protocol must also be provided in the manuscript.

Field-specific reporting

Please select the one below that is the best fit for your research. If you are not sure, read the appropriate sections before making your selection.

☒ Life sciences ☐ Behavioural & social sciences ☐ Ecological, evolutionary & environmental sciences

For a reference copy of the document with all sections, see nature.com/documents/nr-reporting-summary-flat.pdf

Life sciences study design

All studies must disclose on these points even when the disclosure is negative.

Sample size	The datasets comprised of 14 esophageal mucosal tissues suspected of Barrett's neoplasia from 10 patients. No sample-size calculations were performed because this pilot study was exploratory and designed as an initial technical proof of concept.
Data exclusions	No data were excluded from the analyses.
Replication	Data acquisition was performed multiple times on tissues from 2 patients to ensure correct device functioning. All attempts at replication were successful.
Randomization	The study is a technical proof-of-concept, so randomization was not relevant.
Blinding	The gastrointestinal pathologist was blinded to imaging results. Participants in the validation study were blinded to imaging results and histopathology.

Reporting for specific materials, systems and methods

We require information from authors about some types of materials, experimental systems and methods used in many studies. Here, indicate whether each material, system or method listed is relevant to your study. If you are not sure if a list item applies to your research, read the appropriate section before selecting a response.

Materials & experimental systems

n/a	Involved in the study	
☐	☒	Antibodies
☒	☐	Eukaryotic cell lines
☒	☐	Palaeontology and archaeology
☐	☒	Animals and other organisms
☒	☐	Clinical data
☒	☐	Dual use research of concern
☒	☐	Plants

Methods

n/a	Involved in the study	
☒	☐	ChIP-seq
☒	☐	Flow cytometry
☒	☐	MRI-based neuroimaging

Antibodies

Antibodies used

For CD31 immunohistochemistry on paraffin-embedded sections:
Rabbit anti-CD31 (1:50; Abcam, AB28364)

Validation

The antibody, rabbit anti-CD31, was commercially available and therefore validated by previous investigators. Certificates of analysis for the approved applications and relevant references are provided on manufacturer's website (https://www.abcam.com/products/primary-antibodies/cd31-antibody-ab28364.html). It is a polyclonal antibody that recognizes human CD31 and designed for CD31 immunohistochemistry on paraffin-embedded sections.

Animals and other research organisms

Policy information about studies involving animals; ARRIVE guidelines recommended for reporting animal research, and Sex and Gender in Research

Laboratory animals

We conducted ex vivo and in vivo esophageal imaging on 2 cross-bred 6-month-old swines (German Landrace with minipig).

Wild animals

This study did not involve wild animals.

Reporting on sex

Both animals were female. Sex-based analysis is not relevant to this study as there is no evidence that sex impacts the anatomical and morphological features of esophagus.

Field-collected samples

This study did not involve samples collected from the field.

Ethics oversight

An ethics protocol (ROB-55.2-2532.Vet_02-18-33) was approved and granted by the Ethics Committee of Regierung von Oberbayern for the ex vivo and in vivo study.

Note that full information on the approval of the study protocol must also be provided in the manuscript.

Plants

Seed stocks

Report on the source of all seed stocks or other plant material used. If applicable, state the seed stock centre and catalogue number. If plant specimens were collected from the field, describe the collection location, date and sampling procedures.

Novel plant genotypes

Describe the methods by which all novel plant genotypes were produced. This includes those generated by transgenic approaches, gene editing, chemical/radiation-based mutagenesis and hybridization. For transgenic lines, describe the transformation method, the number of independent lines analyzed and the generation upon which experiments were performed. For gene-edited lines, describe the editor used, the endogenous sequence targeted for editing, the targeting guide RNA sequence (if applicable) and how the editor was applied.

Authentication

Describe any authentication procedures for each seed stock used or novel genotype generated. Describe any experiments used to assess the effect of a mutation and, where applicable, how potential secondary effects (e.g. second site T-DNA insertions, mosiacism, off-target gene editing) were examined.
